# Reasons for nonuse of contraceptive methods by women with demand for contraception not satisfied: an assessment of low and middle-income countries using demographic and health surveys

**DOI:** 10.1186/s12978-019-0805-7

**Published:** 2019-10-11

**Authors:** Laísa Rodrigues Moreira, Fernanda Ewerling, Aluisio J. D. Barros, Mariangela Freitas Silveira

**Affiliations:** 1 Postgraduate Program in Epidemiology, Federal University of Pelotas, Marechal Deodoro, 1160 - 3rd floor. Centro, Pelotas, 96020-220 Brazil; 2 International Center for Equity in Health, Federal University of Pelotas, Pelotas, Brazil

**Keywords:** Contraception, Family planning, Reproductive health, Socioeconomic factors, Health inequalities

## Abstract

**Background:**

Nonuse of contraceptive methods by women in need of contraception may impact their sexual and reproductive health. The aim of this study was to describe the reasons for nonuse of contraception among women with demand for contraception not satisfied in low and middle-income countries (considering both overall countries and various subgroups of women).

**Methods:**

We used the latest Demographic and Health Survey data from 47 countries. A descriptive analysis of the reasons for nonuse of contraceptive methods was performed among sexually active women with demand for contraception not satisfied. The prevalence of each reported reason was also evaluated according to marital status, woman’s age and schooling, area of residence, wealth index, and parity. Wealth-related absolute inequality for each reason was also evaluated using the Slope Index of Inequality. A pro-rich inequality pattern means that the reason is more prevalent among the richest women while a pro-poor means the reason is more common among the poorest ones.

**Results:**

On average, 40.9% of women in need of contraception were not using any contraceptive methods to avoid pregnancy. Overall, the most prevalent reasons for nonuse of contraceptives were “health concerns” and “infrequent sex,” but the prevalence of each reason varied substantially across countries. Nonuse due to “opposition from others” was higher among married than unmarried women; in turn, the prevalence of nonuse due to “lack of access” or “lack of knowledge” was about two times higher in rural areas than in urban areas. Women with less schooling more often reported nonuse due to “lack of access.” Pro-rich inequality was detected for reasons “health concerns,” “infrequent sex,” and “method-related”, while the reasons “other opposed,” “fatalistic,” “lack of access,” and “lack of knowledge” were linked to patterns of pro-poor inequality.

**Conclusions:**

Family planning promotion policies must take into account the different reasons for the nonuse of contraceptive methods identified in each country as well as the contextual differences regarding women of reproductive age (such as social norms and barriers that prevent women from accessing and using contraceptives).

## Plain English summary

The nonuse of contraceptive methods by women who need contraception may impact the lives of these women and the planning of their families. One example is unintended pregnancy and its possible implications. Research has identified several reasons for the nonuse of contraceptive methods. However, studies have mostly only focused on women who are married or who live with a partner. In this study, we included all sexually active women in each country; doing so allowed a more comprehensive overview of the reasons for why women whose demands for contraception are not satisfied do not use contraceptive methods. We presented the differences between the reasons for nonuse of contraceptives reported by partnered and unpartnered women. Data from 47 low and middle-income countries were obtained from the Demographic and Health Survey (DHS) Program for analysis. The results showed that, on average, about four out of ten women in need of contraception were not using any contraceptive methods. There was great variation among countries in the reasons reported. Overall, the most common reasons for nonuse were “health concerns” and “infrequent sex.” Nonuse due to “opposition from others” was more common among married women. It was noteworthy that wealthier women often mentioned “health concerns,” “infrequent sex,” and “method-related” as reasons for not using contraceptives. The present research is important in helping professionals from various fields understand why women who need contraception do not use contraceptive methods so as to offer guidance and provide adequate care.

## Background

The nonuse of contraceptive methods by people who need contraception has been associated with potential implications at the individual, familial, community, and global levels. Sustainable Development Goals [1], as well as the proposal of maternal and child health indicators [2], have been essential in increasing the visibility of this topic and highlighting points for improvement. In addition, several agencies and organizations around the world have engaged in global efforts to finance actions that promote family planning, especially in low and middle-income countries (LMIC) [3–5].

However, despite these efforts, the sexual and reproductive rights of women are not always respected. Although there is evidence of progressive increases in the use of contraceptive methods [6], many women still face various barriers to contraceptive use [7]. It is estimated that 214 million women in LMIC in need of contraception do not use any modern contraceptive methods [8]. Considering that all women, as advocated by the Sustainable Development Goals, should have access to sexual and reproductive health services, understanding the reasons behind why this need remains unfulfilled is essential for action planning.

An investigation performed by Sedgh and Hussain [9] on 51 LMIC showed that among married women, the most frequent reasons for nonuse of contraceptives were low frequencies of sexual relations, and fears of side effects and potential health risks. However, a major limitation of that study is the absence of unmarried, sexually active women in the study sample. The reasons for nonuse are likely very different between married and unmarried women, and this is possibly linked to social barriers and other difficulties related to access to contraceptives that unmarried women face. However, literature concerning family planning lacks information regarding reasons, reported by the women themselves, for not using contraception. As discussed by Sedgh and Hussain [9], the analysis of the reasons for nonuse in different population subgroups may provide relevant information to support the design of specific initiatives. Added to that, there is increasing evidence in scientific literature of the importance of using measures that are not only expressed as aggregate data [10], that is, of evaluating the specificities of each country and the possible inequality contexts.

Considering the situation, the present work aimed to address this gap in knowledge. In addition to evaluating the reasons for nonuse of contraceptive methods by sexually active women with demand for contraception not satisfied in LMIC, subgroup analyses of the reasons for nonuse were performed to investigate possible barriers and to propose strategic priorities to improve women’s sexual and reproductive health. Furthermore, the analyses of inequalities might identify what strategies may be more suitable for each subgroup.

## Methods

We analyzed data from the Demographic and Health Surveys (DHS) Program for 47 countries. The study focused on the most recent surveys, with data collected in and after 2010, to provide a current overview of the topic investigated. The Multiple Indicator Cluster Surveys (MICS) were not included, since the data of interest could not be retrieved from these surveys in a manner that allowed for comparison. DHS are standardized surveys that use a cross-sectional design to collect data from LMIC. All surveys use similar questionnaires, methodology, and sampling strategies, which ensures the comparability of results. In general, the areas of interest are initially identified through censuses or other procedures. After that, households are selected based on pre-established methodological steps [11, 12]. The present analysis included the latest available DHS with information on demands for family planning and covered all sexually active women of reproductive age (15–49 years) in each country. Six surveys that did not contain information on sexually active women living without a partner were excluded (Afghanistan 2015, Bangladesh 2014, Egypt 2014, Jordan 2012, Pakistan 2012, Yemen 2013).

### Definitions and outcomes

The study’s primary outcomes were the reasons reported by sexually active women of reproductive age (15–49 years) (with demand for contraception not satisfied) for not using contraceptive methods. The indicator Demand for Contraception Not Satisfied utilizes the number of women who are not using any contraceptive methods in its numerator and the number of women of reproductive age (15–49 years) in need of contraception in its denominator. In other words, the study included women who were in need of contraception but did not use any contraceptive methods. Women in need of contraception were defined as those who were sexually active and fertile, but did not intend to become pregnant in the next 2 years or did not know if or when they intended to become pregnant [2, 11, 13]. Sexually active women were defined as those who were married or lived with a partner and those who were not married but had sexual intercourse in the 30 days prior to the survey. Infertile women excluded from the study were defined as those who: 1) had never had a period or were amenorrhoeic in the 6 months before the survey even though they were not in the postpartum period, 2) were hysterectomized, and 3) had been married or in a stable relationship for 5 years or more but did not become pregnant despite a lack of contraceptive use [11, 14]. Although pregnant women who considered their pregnancies untimely or had not desired their pregnancies are generally classified as in need of contraception [11], they were also excluded from this study because being pregnant is one of the filters for the questions regarding the reasons for nonuse of contraception.

The reasons for nonuse were evaluated using the following question: “You have said that you do not want any (more) children. Can you tell me why you are not using a method to prevent pregnancy? Any other reason?” The reasons for not using contraceptive methods were grouped into eight broad groups of reasons (see Table 1 for details): 1) “respondent opposed” (i.e., the woman herself opposed contraceptive use), 2) “other opposed,” 3) “lack of knowledge,” 4) “health concerns,” 5) “lack of access,” 6) “method-related” (inconvenient to use), 7) “fatalistic” (or up to God, meaning that the woman feels that pregnancies are determined by fate), and 8) “infrequent sex.” Because women could report more than one reason, each response category was analyzed separately.

**Table 1:** Operational definition of the reasons for not using contraceptive methods

Outcomes (reasons) definition	Reasons included
1) Respondent opposed	Respondent opposed
2) Other opposed	Husband or partner opposed
	Others opposed
	Religious prohibition
3) Lack of knowledge	Knows no method
	Knows no source
4) Health concerns	Health concern
	Fear of side effects
	Interferes with bodily processes
5) Lack of Access	Too far
	Costs too much
	No method available
	Preferred method not available
6) Method-related	Method is inconvenient to use
7) Fatalistic	Fatalistic
8) Infrequent sex	Not having sex
	Infrequent sex

### Statistical analysis

Descriptive analyses were used to identify the main reasons for the nonuse of contraceptive methods among women whose demands for contraception were not satisfied in the various LMIC. When analyzing the reasons for nonuse, the countries were ranked according to the level of demand for contraception not satisfied: less than 30.0%, 30.0% to 50.0%, and more than 50.0%. Considering these coverage levels, the reasons for nonuse were also evaluated according to the following stratification variables:

- marital status (married/union; unmarried sexually active);
- woman’s age (15 to 19; 20 to 34; 35 to 49 years);
- area of residence (urban or rural);
- woman’s education (none; primary/elementary school; secondary or higher);
- parity defined as the number of live births (0; 1–2; 3 or more);
- wealth index (in quintiles, with the first corresponding to the poorest 20% and the fifth to the wealthiest 20% in each country). The wealth index is calculated using principal component analyses which take into account characteristics of the household, ownership of selected assets, and educational attainment of the head of the family [15, 16].

In addition, inequality analyses were performed using the Slope Index of Inequality (SII). Instead of calculating the simple absolute difference in the prevalence of the outcome of interest in the wealthiest vs. poorest quintile, the SII measures the difference in predicted coverage using a statistical model for these quintiles. In other words, the SII takes into account the prevalence of the outcome of interest in the five wealth quintiles to estimate the absolute difference between the wealthiest and the poorest quintile [15, 16]. All statistical analyses were performed using the STATA 13.1 statistical package [17] and took into account the sample weights of surveys through the command “svy.”

## Results

Considering the 47 countries analyzed, the mean prevalence of demand for contraception not satisfied was 40.9% (95% CI: 38.9–43.0). Seventeen countries had a prevalence of demand for contraception not satisfied higher than 50%, and in five of these countries (Angola, Mali, Gambia, Guinea, and Chad), the prevalence was 70% or higher. The Republic of Chad had the highest prevalence (79.7%, 95% CI: 77.2–82.1) (Table 2). The two countries with the lowest prevalence of demand for contraception not satisfied were Colombia (8.6%, 95% CI: 8.1–9.2) and Honduras (12.8%, 95% CI: 12.1–13.5), both located in Latin America and the Caribbean.

**Table 2:** Overall description (countries information, prevalence of demand for contraception not satisfied and 95% CI)

Country	Year	ISO	Region	Income group	Demand for contraception not satisfied % (95% CI)
Prevalence of demand for contraception not satisfied < 30.0%					
Colombia	2015	COL	Latin America and Caribbean	Upper-middle income	8.6 (8.1–9.2)
Honduras	2011	HND	Latin America and Caribbean	Lower-middle income	12.8 (12.1–13.5)
Zimbabwe	2015	ZWE	Eastern and Southern Africa	Low income	14.0 (12.8–15.4)
Dominican Republic	2013	DOM	Latin America and Caribbean	Upper-middle income	14.6 (13.0–16.3)
Indonesia	2012	IDN	East Asia and the Pacific	Lower-middle income	15.6 (14.8–16.4)
Armenia	2015	ARM	Europe and Central Asia	Lower-middle income	17.9 (16.2–19.6)
Cambodia	2014	KHM	East Asia and the Pacific	Low income	18.2 (16.9–19.6)
Guatemala	2014	GTM	Latin America and Caribbean	Lower-middle income	18.7 (17.7–19.8)
India	2015	IND	South Asia	Lower-middle income	19.4 (19.1–19.6)
Namibia	2013	NAM	Eastern and Southern Africa	Upper-middle income	20.8 (19.2–22.4)
Lesotho	2014	LSO	Eastern and Southern Africa	Lower-middle income	23.1 (21.3–25.1)
Kenya	2014	KEN	Eastern and Southern Africa	Lower-middle income	23.6 (22.3–25.0)
Myanmar	2015	MMR	East Asia and the Pacific	Lower-middle income	23.8 (22.3–25.3)
Philippines	2017	PHL	East Asia and the Pacific	Lower-middle income	24.3 (23.0–25.6)
Malawi	2015	MWI	Eastern and Southern Africa	Low income	25.2 (24.2–26.3)
Congo Brazzaville	2011	COG	West and Central Africa	Lower-middle income	26.2 (24.2–28.2)
Rwanda	2014	RWA	Eastern and Southern Africa	Low income	27.7 (26.2–29.3)
Prevalence of demand for contraception not satisfied 30.0–50.0%					
Nepal	2016	NPL	South Asia	Low income	31.1 (29.6–32.5)
Zambia	2013	ZMB	Eastern and Southern Africa	Lower-middle income	32.3 (30.8–33.8)
Kyrgyzstan	2012	KGZ	Europe and Central Asia	Low income	33.7 (31.3–36.1)
Tanzania	2015	TZA	Eastern and Southern Africa	Low income	35.9 (34.0–37.8)
Ethiopia	2016	ETH	Eastern and Southern Africa	Low income	38.1 (35.1–41.3)
Gabon	2012	GAB	West and Central Africa	Upper-middle income	41.5 (38.0–45.0)
Uganda	2016	UGA	Eastern and Southern Africa	Low income	41.8 (40.2–43.4)
Senegal	2017	SEN	West and Central Africa	Low income	44.1 (41.9–46.3)
Mozambique	2015	MOZ	Eastern and Southern Africa	Low income	44.6 (41.7–47.5)
Tajikistan	2012	TJK	Europe and Central Asia	Low income	45.0 (42.7–47.4)
Cameroon	2011	CMR	West and Central Africa	Lower-middle income	46.4 (44.4–48.5)
Nigeria	2013	NGA	West and Central Africa	Lower-middle income	47.7 (45.8–49.6)
Timor-Leste	2016	TLS	East Asia and the Pacific	Lower-middle income	49.7 (47.0–52.4)
Prevalence of demand for contraception not satisfied > 50.0%					
Burundi	2016	BDI	Eastern and Southern Africa	Low income	50.9 (49.1–52.7)
Sierra Leone	2013	SLE	West and Central Africa	Low income	52.0 (48.9–55.1)
Ghana	2014	GHA	West and Central Africa	Lower-middle income	52.1 (49.6–54.7)
Niger	2012	NER	West and Central Africa	Low income	53.5 (50.6–56.5)
Haiti	2016	HTI	Latin America and Caribbean	Low income	53.9 (52.0–55.7)
Congo Democratic Republic	2013	COD	West and Central Africa	Low income	56.4 (53.0–59.7)
Côte d’Ivoire	2011	CIV	West and Central Africa	Lower-middle income	58.0 (55.0–61.0)
Burkina Faso	2010	BFA	West and Central Africa	Low income	58.8 (56.8–60.8)
Liberia	2013	LBR	West and Central Africa	Low income	59.0 (55.3–62.7)
Togo	2013	TGO	West and Central Africa	Low income	60.8 (58.5–63.1)
Comoros	2012	COM	Eastern and Southern Africa	Low income	61.2 (57.7–64.6)
Benin	2011	BEN	West and Central Africa	Low income	69.6 (68.0–71.2)
Angola	2015	AGO	Eastern and Southern Africa	Upper-middle income	70.7 (67.6–73.7)
Mali	2012	MLI	West and Central Africa	Low income	71.0 (68.6–73.3)
Gambia	2013	GMB	West and Central Africa	Low income	72.9 (69.4–76.1)
Guinea	2012	GIN	West and Central Africa	Low income	75.7 (73.0–78.2)
Chad	2014	TCD	West and Central Africa	Low income	79.7 (77.2–82.1)

“Health concerns” was the top reason for nonuse in 22 out of the 47 countries. In 18 countries (38.3% of the total countries), “infrequent sex” was the most prevalent reason (Table 3). In Gabon, the same prevalence (17.4%) was recorded for both “health concerns” and “infrequent sex,” and the two were also the most common reasons for nonuse in this country. “Other opposed” was the most prevalent reason in three countries (Senegal, Mali, and Guinea). In Gambia, “respondent opposed” was the most prevalent reason for nonuse whereas in Ethiopia and Niger, “fatalistic” was the most common reason (Table 3).

**Table 3:** Reasons for nonuse of contraceptive methods (mean by coverage level, prevalence, and 95% CI)

Country	Health concerns	Infrequent sex	Other opposed	Respondent opposed	Fatalistic	Lack of access	Lack of knowledge	Method-related	N (all reasons)
Demand for contraception not satisfied < 30.0%	23.5	29.9	7.3	5.1	4.5	6.0	1.8	1.6	
Colombia	17.6	26.7	4.6	5.0	Na	4.3	0.7	Na	1566
	(15.0–20.5)	(23.7–30.0)	(3.2–6.5)	(3.7–6.6)		(3.3–5.6)	(0.4–1.3)		
Honduras	19.5	51.0	9.1	4.5	1.4	3.1	0.3	0.5	1116
	(16.9–22.5)	(47.4–54.6)	(7.2–11.3)	(3.4–6.0)	(0.7–2.7)	(2.2–4.4)	(0.1–0.9)	(0.3–1.1)	
Zimbabwe	18.9	41.6	14.0	1.7	0.8	5.5	0 (0–0)	1.7	468
	(14.7–23.9)	(35.7–47.7)	(10.1–19.0)	(0.8–3.8)	(0.3–2.3)	(3.7–8.2)		(0.8–3.3)	
Dominican Republic	28.8	24.0	3.3	10.8	5.4	5.0	0.7	1.2	477
	(22.7–35.7)	(19.6–29.1)	(1.8–6.0)	(7.8–14.8)	(2.8–10.3)	(1.8–13.6)	(0.3–1.6)	(0.6–2.4)	
Indonesia	29.8	22.3	1.8	0.5	2.3	3.6	0.9	4.0	2985
	(27.3–32.5)	(19.8–25.0)	(1.2–2.5)	(0.3–0.8)	(1.5–3.5)	(2.6–4.9)	(0.6–1.4)	(3.0–5.3)	
Armenia	15.0	25.8	5.2	11.7	10.2	0	1.2	2.1	308
	(11.6–19.1)	(21.1–31.1)	(3.3–8.3)	(8.0–16.8)	(7.1–14.4)		(0.3–5.0)	(0.9–4.9)	
Cambodia	36.1	45.5	1.9	3.3	10.9	2.6	1.4	2.1	1195
	(32.8–39.6)	(41.3–49.8)	(1.0–3.7)	(2.0–5.5)	(8.9–13.1)	(1.6–4.0)	(0.8–2.6)	(1.3–3.5)	
Guatemala	17.0	50.3	10.8	6.0	3.2	4.9	3.5	0.8	1769
	(14.9–19.3)	(47.3–53.3)	(9.0–12.9)	(4.8–7.4)	(2.3–4.5)	(3.9–6.2)	(2.5–4.7)	(0.5–1.3)	
India	7.4	31.0	19.7	8.1	9.1	9.8	2.3	1.0	57008
	(7.0–7.7)	(30.4–31.7)	(19.1–20.2)	(7.7–8.5)	(8.7–9.4)	(9.3–10.2)	(2.1–2.6)	(0.8–1.1)	
Namibia	26.3	11.0	9.9	5.9	4.0	15.3	2.4	1.0	527
	(22.0–31.1)	(8.3–14.4)	(7.1–13.5)	(3.9–9.1)	(2.7–6.1)	(12.0–19.4)	(1.3–4.5)	(0.5–2.1)	
Lesotho	11.0	39.5	5.5	1.7	2.7	5.2	0.4	1.7	605
	(8.3–14.5)	(35.0–44.3)	(3.6–8.3)	(0.8–3.7)	(1.5–4.9)	(3.6–7.3)	(0.1–1.8)	(0.9–3.3)	
Kenya	42.0	23.1	9.4	4.2	3.4	2.8	1.7	0.9	1382
	(38.5–45.5)	(20.1–26.3)	(7.6–11.5)	(3.0–5.6)	(2.3–5.1)	(1.6–4.9)	(1.1–2.4)	(0.5–1.6)	
Myanmar	32.6	32.8	1.2	0.9	2.9	4.8	0.4	2.7	1208
	(29.1–36.4)	(29.4–36.4)	(0.7–2.2)	(0.5–1.8)	(1.9–4.4)	(3.1–7.5)	(0.2–1.2)	(1.8–4.1)	
Philippines	26.3	22.3	7.8	10.1	3.7	13.7	0.6	0.3	1878
	(23.8–29.1)	(19.0–26.1)	(6.0–10.2)	(8.2–12.4)	(2.8–4.9)	(11.5–16.2)	(0.3–1.3)	(0.1–0.7)	
Malawi	20.2	25.1	5.3	4.9	4.2	5.7	0.4	2.2	1947
	(18.1–22.5)	(22.5–28.0)	(4.2–6.6)	(3.8–6.2)	(3.2–5.3)	(4.5–7.1)	(0.2–0.9)	(1.5–3.1)	
Congo Brazzaville	17.0	15.1	9.5	5.0	0.4	14.2	12.6	1.4	861
	(13.1–21.8)	(11.6–19.5)	(6.5–13.7)	(2.9–8.5)	(0.1–2.2)	(10.3–19.2)	(9.8–16.0)	(0.4–4.2)	
Rwanda	34.3	20.8	5.7	3.1	7.2	1.7	0.4	2.3	1057
	(31.2–37.5)	(18.2–23.6)	(4.4–7.4)	(2.2–4.3)	(5.7–9.1)	(1.1–2.8)	(0.1–1.0)	(1.5–3.4)	
Demand for contraception not satisfied 30.0–50.0%	21.4	18.4	12.3	8.8	8.8	5.1	3.6	1.8	
Nepal	15.0	18.2	5.3	0.2	0.7	0.9	0.2	1.2	1425
	(12.6–17.8)	(15.5–21.3)	(4.0–7.1)	(0.0–0.8)	(0.4–1.4)	(0.4–1.9)	(0.0–0.6)	(0.6–2.6)	
Zambia	30.1	19.3	10.0	3.3	3.1	5.0	1.0	1.2	1578
	(27.2–33.1)	(17.1–21.7)	(8.2–12.2)	(2.3–4.7)	(2.2–4.3)	(3.9–6.4)	(0.6–1.8)	(0.7–2.1)	
Kyrgyzstan	20.9	17.3	17.1	12.2	8.6	1.2	1.5	0.8	642
	(16.8–25.7)	(14.1–21.1)	(13.1–22.0)	(9.4–15.8)	(6.2–11.9)	(0.5–3.0)	(0.6–3.5)	(0.2–3.0)	
Tanzania	40.3	17.0	16.1	14.6	8.7	3.9	1.3	1.3	1515
	(37.1–43.6)	(14.7–19.6)	(14.0–18.4)	(12.5–16.9)	(7.2–10.5)	(2.9–5.3)	(0.8–2.2)	(0.5–3.1)	
Ethiopia	16.3	9.0	13.3	6.6	23.7	3.5	1.0	1.7	1316
	(12.8–20.7)	(6.9–11.6)	(10.2–17.1)	(4.6–9.2)	(20.1–27.6)	(2.2–5.4)	(0.4–2.3)	(1.0–3.0)	
Gabon	17.4	17.4	11.0	5.7	2.4	6.2	8.5	4.7	1063
	(13.9–21.6)	(13.8–21.7)	(8.2–14.5)	(3.7–8.7)	(1.3–4.6)	(4.6–8.5)	(6.4–11.1)	(2.9–7.4)	
Uganda	21.3	19.8	10.8	5.7	6.9	2.5	0.3	0.9	2454
	(19.4–23.4)	(17.9–21.9)	(9.5–12.3)	(4.8–6.8)	(5.8–8.1)	(1.9–3.3)	(0.1–0.6)	(0.6–1.5)	
Senegal	15.0	19.0	21.1	12.2	9.6	2.7	0.8	0.3	1641
	(13.0–17.4)	(16.6–21.6)	(18.5–24.0)	(10.2–14.5)	(7.9–11.7)	(1.9–3.7)	(0.5–1.5)	(0.1–0.9)	
Mozambique	9.6	19.9	7.2	6.5	9.7	3.7	5.0	2.4	809
	(7.4–12.5)	(16.8–23.4)	(4.8–10.6)	(4.7–9.0)	(7.7–12.1)	(2.4–5.5)	(3.3–7.6)	(1.5–3.8)	
Tajikistan	14.5	27.8	12.8	24.8	10.9	2.4	0.3	1.0	1118
	(12.0–17.4)	(24.4–31.5)	(10.4–15.7)	(21.5–28.3)	(8.8–13.6)	(1.5–3.8)	(0.1–0.9)	(0.5–1.9)	
Cameroon	21.9	29.3	12.9	7.4	1.5	9.5	15.2	2.5	1873
	(19.6–24.4)	(26.4–32.3)	(11.1–14.9)	(6.2–8.9)	(0.9–2.4)	(7.8–11.6)	(12.8–18.1)	(1.8–3.5)	
Nigeria	19.5	17.8	17.9	15.3	7.4	5.1	10.1	2.9	3459
	(17.7–21.4)	(16.4–19.4)	(16.2–19.7)	(13.6–17.2)	(6.3–8.6)	(4.2–6.1)	(8.6–11.7)	(2.3–3.7)	
Timor-Leste	35.8	7.0	4.0	0	21.1	19.3	1.9	2.4	759
	(31.5–40.2)	(4.9–9.9)	(2.4–6.5)		(17.2–25.5)	(16.2–22.8)	(1.0–3.4)	(1.3–4.3)	
Demand for contraception not satisfied > 50.0%	21.3	16.2	14.8	10.5	7.2	6.9	6.9	2.1	
Burundi	33.0	11.7	27.2	7.9	2.5	1.9	0.3	0.8	2196
	(30.7–35.3)	(10.2–13.4)	(24.9–29.6)	(6.6–9.5)	(1.8–3.4)	(1.4–2.8)	(0.1–0.8)	(0.5–1.4)	
Sierra Leone	13.4	24.1	10.2	9.1	9.8	7.4	2.9	2.2	2173
	(11.7–15.4)	(21.1–27.3)	(8.6–12.1)	(7.2–11.5)	(8.1–11.8)	(5.9–9.2)	(2.1–4.1)	(1.6–3.0)	
Ghana	46.8	19.2	7.7	7.5	5.3	5.7	1.2	5.4	1328
	(43.0–50.7)	(16.2–22.5)	(5.9–10.1)	(4.8–11.4)	(4.2–6.8)	(4.3–7.6)	(0.7–2.0)	(4.0–7.1)	
Niger	6.2	19.1	11.7	5.6	21.5	6.9	5.8	4.1	1251
	(4.8–7.9)	(16.1–22.4)	(9.6–14.2)	(4.3–7.3)	(18.1–25.3)	(5.3–8.9)	(4.2–7.8)	(2.9–5.9)	
Haiti	40.2	14.7	11.4	20.4	11.1	2.9	0.1	2.0	2855
	(37.6–42.8)	(13.0–16.6)	(9.8–13.3)	(18.1–22.9)	(9.7–12.8)	(2.1–4.1)	(0.0–0.4)	(1.4–2.7)	
Congo Democratic Republic	18.4	19.6	17.4	11.3	3.1	8.6	14.5	1.5	2653
	(15.9–21.2)	(17.3–22.2)	(15.3–19.7)	(9.7–13.2)	(2.2–4.2)	(6.7–10.9)	(11.3–18.5)	(1.1–2.3)	
Côte d’Ivoire	24.2	15.1	14.0	9.7	2.8	7.3	15.5	1.9	1510
	(21.4–27.2)	(12.7–17.9)	(11.8–16.4)	(6.8–13.9)	(1.8–4.2)	(5.4–9.8)	(12.5–19.0)	(1.1–3.0)	
Burkina Faso	14.7	22.8	18.3	6.3	5.1	14.5	3.4	0.8	2917
	(13.1–16.4)	(20.8–24.9)	(16.5–20.3)	(5.3–7.4)	(4.1–6.4)	(12.8–16.4)	(2.6–4.5)	(0.5–1.2)	
Liberia	27.3	15.4	11.7	15.2	7.4	6.4	4.1	6.0	1863
	(23.8–31.1)	(13.0–18.2)	(9.1–15.0)	(12.0–19.1)	(5.8–9.4)	(5.1–8.2)	(3.1–5.6)	(4.5–7.9)	
Togo	34.4	19.6	9.2	8.6	11.3	6.2	4.2	2.7	1787
	(31.4–37.5)	(17.5–21.9)	(7.4–11.2)	(6.8–10.8)	(9.3–13.6)	(5.1–7.6)	(3.0–5.7)	(1.9–3.8)	
Comoros	29.3	11.2	14.5	6.5	0.2	16.3	0.3	1.3	719
	(24.6–34.6)	(8.2–15.0)	(11.5–18.1)	(4.6–9.3)	(0.1–1.0)	(12.7–20.6)	(0.1–1.1)	(0.6–2.5)	
Benin	19.8	15.8	14.2	5.1	3.0	11.8	10.6	2.6	2361
	(17.7–22.1)	(14.0–17.8)	(12.6–15.9)	(4.2–6.2)	(2.3–3.9)	(10.5–13.3)	(9.3–11.9)	(2.0–3.4)	
Angola	13.3	6.8	10.0	9.6	9.7	2.1	7.5	1.8	1971
	(11.1–15.9)	(5.5–8.4)	(7.5–13.2)	(7.6–12.1)	(8.2–11.5)	(1.5–3.1)	(6.0–9.5)	(1.1–3.0)	
Mali	9.3	12.5	26.4	8.4	5.9	7.3	10.3	0.8	1527
	(7.4–11.5)	(10.4–14.9)	(22.7–30.5)	(6.9–10.3)	(4.5–7.7)	(5.6–9.5)	(8.4–12.6)	(0.4–1.7)	
Gambia	12.6	14.8	17.2	25.4	14.1	1.9	6.4	0.5	1482
	(10.0–15.9)	(12.4–17.5)	(13.9–21.0)	(21.2–30.0)	(11.3–17.6)	(1.2–2.9)	(4.1–9.9)	(0.2–1.3)	
Guinea	11.0	15.1	15.5	11.7	6.2	6.0	14.7	0.3	1315
	(8.7–13.8)	(12.7–17.9)	(13.3–18.0)	(9.4–14.6)	(4.2–9.0)	(4.4–8.1)	(12.0–17.9)	(0.1–0.9)	
Chad	7.8	18.0	14.7	10.9	3.0	3.6	15.4	0.4	1918
	(6.3–9.6)	(15.7–20.5)	(12.7–17.0)	(9.2–13.0)	(2.0–4.4)	(2.7–4.8)	(13.2–17.9)	(0.2–0.8)	

The prevalence of the reasons reported were also different according to the level of demand for contraception not satisfied (Table 3). On average, “health concern” and “infrequent sex” were the most common reasons for all levels of demand not satisfied, but the prevalence of these reasons was higher among countries with demand not satisfied below 30%. All the other reasons tended to be more prevalent among countries with higher demand not satisfied.

It is important to note that the most common reasons for nonuse of contraception varied greatly between countries. In the five countries with demand not satisfied of 70% or more, the most prevalent reasons for nonuse were: Angola - “health concerns” (13.3%, 95% CI: 11.1–15.9), Mali - “other opposed” (26.4%, 95% CI: 22.7–30.5), Gambia - “respondent opposed” (25.4%, 95% CI: 21.2–30.0), Guinea - “other opposed” (15.5%, 95% CI: 13.3–18.0), and Chad - “infrequent sex” (18.0%, 95% CI: 15.7–20.5).

**Fig. 1:**
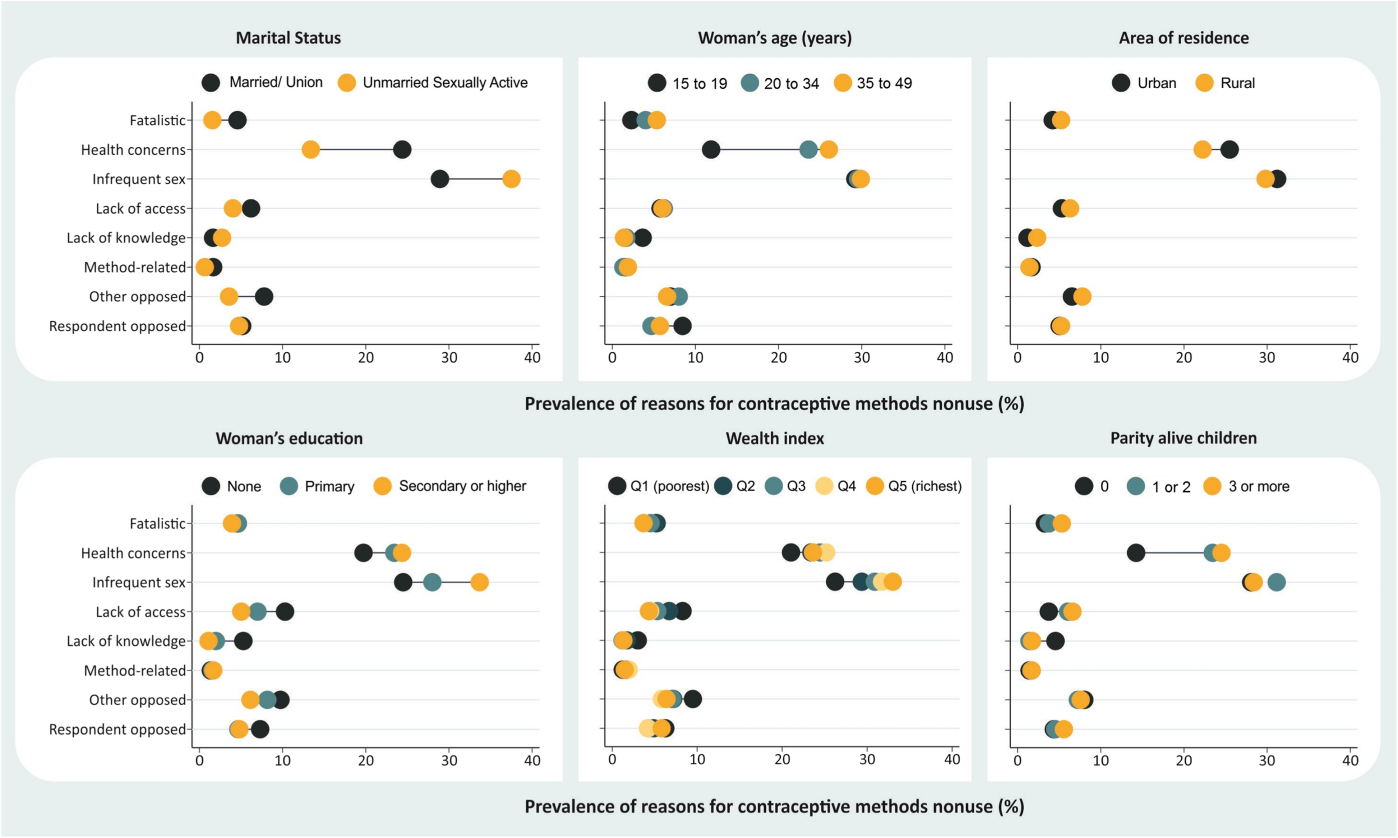
Reasons for nonuse of contraceptive methods according to stratifiers in countries with demand for contraception not satisfied < 30.0%

**Fig. 2:**
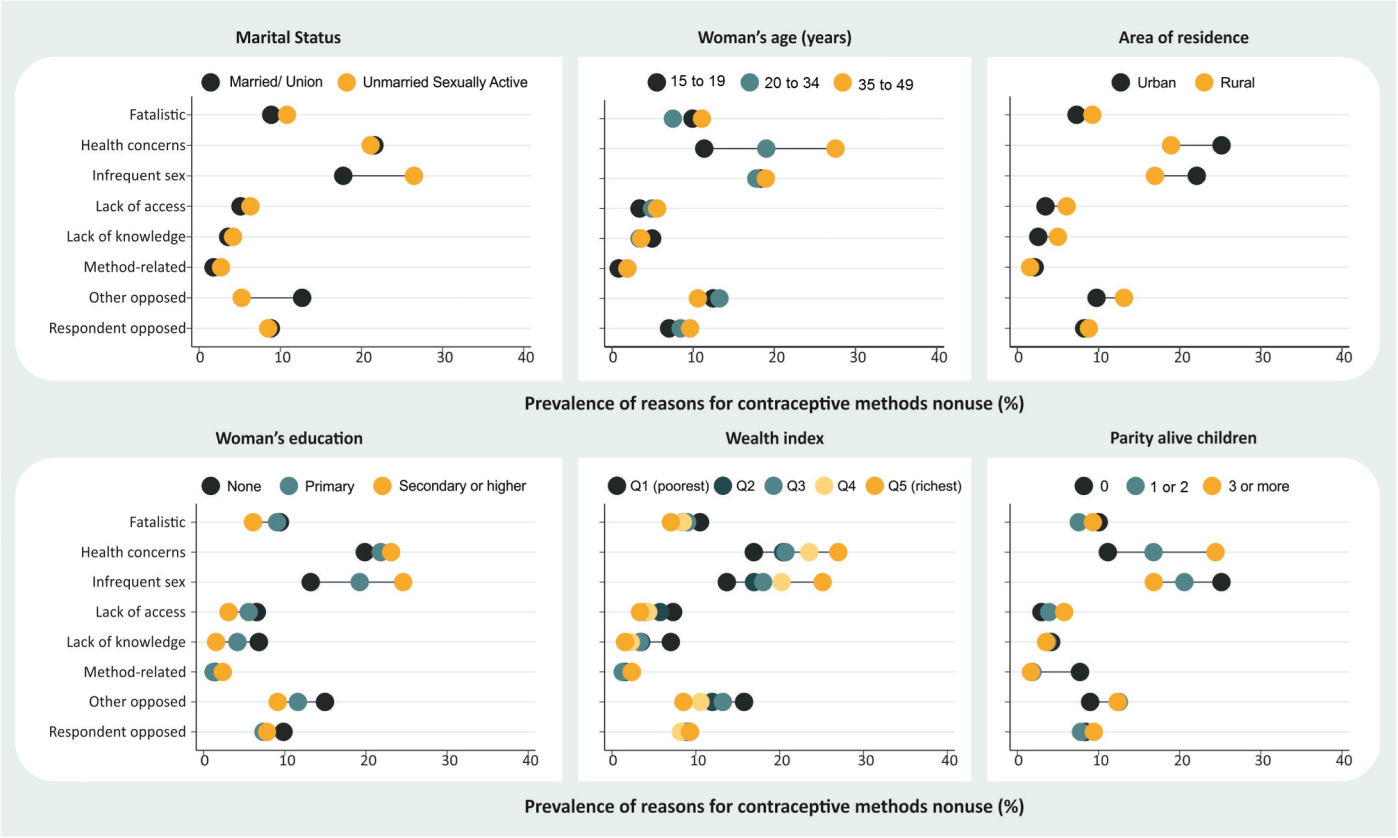
Reasons for nonuse of contraceptive methods according to stratifiers in countries with demand for contraception not satisfied between 30.0 and 50.0%

**Fig. 3:**
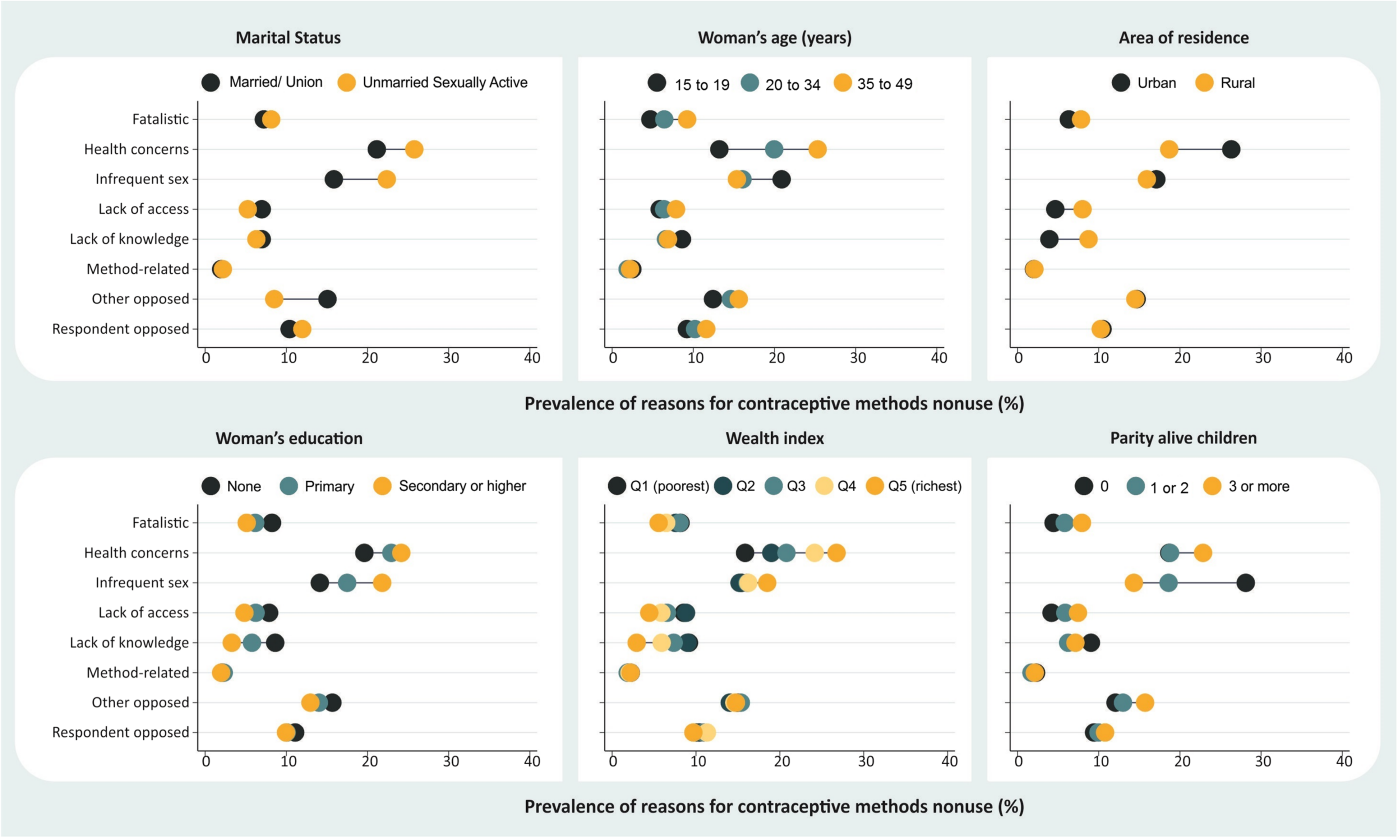
Reasons for nonuse of contraceptive methods according to stratifiers in countries with demand for contraception not satisfied > 50.0%

Stratification provided an overview of the situation in the countries according to different subgroups of women. These analyses are presented by level of demand for contraception not satisfied: countries with demand not satisfied below 30% (Fig. 1), from 30% to 50% (Fig. 2), and above 50% (Fig. 3). We also provide the stratified estimates for each reason by country in the supplementary material (see Additional files 1, 2, 3, 4, 5, 6, 7 and 8). These analyses reveal important differences on why some women do not use contraception.

Nonuse due to “health concerns” was more frequently reported among women who were older, had higher levels of education, had more wealth, were married or living with a partner, had at least one child, and among those living in urban areas. “Infrequent sex,” the second most reported reason, was higher among those who had higher levels of education, had more wealth, were unmarried, and had no children. In all the coverage levels assessed, a difference close to eight percentage points was observed for the comparison between married and unmarried women in “infrequent sex,” with the latter being more likely to mention this reason (Figs. 1, 2 and 3).

There were no marked inequalities in the reporting of nonuse due to “respondent opposed.” On the other hand, nonuse as a result of “other opposed” occurred mostly among married women rather than unmarried women in all coverage levels assessed. However, the prevalence of this reason was higher among countries with higher demand not satisfied. For those with demand not satisfied of > 50%, “opposition by others” was reported by 15.1% of married women and 8.5% of unmarried women (Fig. 3) while for countries with demand not satisfied of < 30%, this reason was reported by 7.8% of married women and 3.6% of unmarried women (Fig. 1). In countries with demand not satisfied between 30 and 50%, there were higher inequalities observed in terms of wealth and education; poorer and less educated women were more likely to report this reason (Fig. 2).

Nonuse due to “fatalistic,” “lack of access,” and “lack of knowledge” were more frequently reported by women in the poorer groups, those with lower education levels, and those who lived in rural areas (Figs. 1, 2 and 3). “Fatalistic” as the reason for nonuse was also systematically higher among older women and those with higher parity. “Lack of knowledge” was also about twice higher in rural areas than in urban areas for all levels of demand for contraception not satisfied evaluated. Nonuse due to “lack of access” was also higher in rural areas than in urban areas for all levels of demand for contraception not satisfied (< 30%, 30–50% and > 50%), corresponding to, respectively, 5.3%, 3.5%, and 4.6% in urban areas, and 6.3%, 6.1%, and 8.0% in rural areas (Figs. 1, 2 and 3).

Regarding the inequality among the reasons for not using contraceptive methods by country, determined by the SII, the results clearly show that nonuse due to “health concerns,” “infrequent sex,” and “method-related” showed pro-rich inequality patterns (SII > 0) in most countries, which means that these reasons were more prevalent among the richest women (see Additional file 9). On the other hand, the reasons “other opposed,” “fatalistic,” “lack of access,” and “lack of knowledge” were linked to patterns of pro-poor inequality (SII < 0), which means that these reasons were more prevalent among the poorest women.

## Discussion

This study evaluated the reasons for nonuse of contraception among women with demand for contraception not satisfied in 47 LMIC. The mean prevalence of demand for contraception not satisfied in this group of countries was 40.9% (95% CI: 38.9–43.0). Regarding the reasons for nonuse by women with demand for contraception not satisfied, it is noteworthy that “health concerns” and “infrequent sex” were the most prevalent reasons in many countries. Five countries, all of which are located in Africa (Angola, Chad, Gambia, Guinea and Mali), presented prevalence of demand not satisfied of > 70.0%.

In West and Central Africa, where many of the low-income countries analyzed are located, a study identified inequalities and a higher probability of long-term contraceptive use by the wealthier women in this region than by the poorer women [18]. This underscores the need to empower women and the importance of learning about the unique characteristics of each region, for example.

Conversely, Colombia (8.6%, 95% CI: 8.1–9.2) and Honduras (12.8%, 95% CI: 12.1–13.5), located in Latin American and the Caribbean, had the lowest prevalence of demand for contraception not satisfied. The literature on Latin America and the Caribbean describes a decline in fertility rate over time [19] as well as other demographic changes, such as aging populations [20]. In this context, reproductive health policies, among other strategies, play an important role in family planning [21]. Some advances also may be attributed to the availability of a variety of contraceptive methods [20]. It is possible that these various processes together may explain the low levels of demand for contraception not satisfied identified in Colombia and Honduras. However, it is important to highlight that the low prevalence of demand for contraception not satisfied in these countries do not reflect an ideal scenario, although some of these countries have low levels of demand not satisfied, the optimal prevalence for this indicator would be zero. All women in need of contraceptives should have access to them.

Practicing contraception does have side effects, and nonuse due to fear of these consequences was addressed in the reason “health concerns.” Conversely, one possible result of not using any contraceptive methods is unintended pregnancy [22]. It should be noted that, besides the associated stigma, unintended pregnancy may have other consequences for the woman and her family of which the general population is often unaware. Such consequences may include negative health outcomes for the woman (morbidity and mortality) and the child (for example, impact on prenatal care and breastfeeding) as well as social costs [23–25]. However, it should be noted that unintended pregnancies are not always regarded as negative outcomes by women and their families, which shows the importance of contextual variables [26]. Nevertheless, studies also suggest that the possible side effects of contraceptive use are few compared to the possible risks resulting from some types of pregnancies [27–29].

Women in the highest wealth quintile had a higher mean prevalence of nonuse due to “health concern.” This raises the question of whether these women have access to a wide range of contraceptive methods or whether this access is restricted to only a few contraceptive options that may not meet individual demands. In this study, we showed that some participants mentioned “method-related” reasons for not using contraceptives which may indicate dissatisfaction with the existing methods of contraception; this supports the need for a wider range of contraceptive options, as pointed out by Barot [30]. In addition, health care professionals need to provide evidence-based information about the methods of contraception and listen to women’s beliefs and opinions so that women can make informed decisions about the most appropriate contraceptive methods for them.

Women who reported “infrequent sex” as a reason for nonuse may consider themselves less likely to get pregnant, as suggested by Sedgh and Hussain [9] and Barot [30]. Over time, women have taken on new roles in society, and there is evidence of declining unmet contraceptive needs [6, 31]. Work opportunities may sometimes lead to geographical relocation [9, 32, 33], which may then cause couples to live apart. In this situation, the professional advantage obtained may come at a personal cost with regards to women’s reproductive health decisions. These aspects may explain, at least in part, some of our results (for example, why women who were in the highest wealth quintile (fifth quintile) and had more schooling had a higher mean prevalence of nonuse due to “infrequent sex”). It is important to provide these women information on the functioning of the reproductive system and the importance of contraceptive use, and offer them support for reproductive health decisions. Long-acting reversible contraceptives may be a good alternative for these women.

Some women avoid using contraceptives due to “other opposition” which can be their partners, other people from their families or communities, or even their religions. Women sometimes face obstacles in their relationships with their partners (for example, difficulty in negotiating contraceptive use), and this can be inferred from the stratified analysis since in general, the mean prevalence of this reason was higher among married women than among unmarried women. This is striking, as all women should have the right to make their own reproductive decisions. This also highlights the need to empower women, as doing so will allow them to have autonomy over their own bodies and lives and make informed decisions regarding contraception [34].

It is possible that cultural and personal issues, which were not captured in the health surveys, may also be involved in women’s decisions to use or not use contraceptive methods. Some women are engaged in homoaffective relationships, which is another aspect that is not evaluated in the surveys. In this case, there would be no concerns regarding using contraception to prevent pregnancy, although the use of condoms, for example, would be essential in preventing sexually transmitted infections. In regard to women who reported a “fatalistic” or “respondent opposed” reason, it is possible that this involves beliefs or other personal issues that require further investigation.

In countries where nonuse due to “lack of access” is high, investments made to increase the availability of contraceptive methods is essential. Beyond access, it is also imperative that health care professionals are prepared to provide information on the specific characteristics of existing contraceptive methods to ensure that people can choose the method that best fits their individual needs [23, 35, 36]. However, it is noteworthy that some women face a variety of barriers [7, 37] when attempting to fulfill their reproductive health needs.

Some limitations of the present study must be addressed. First, since countries without information on unmarried, sexually active women were excluded, some regions may have been underrepresented. In other words, the group of countries that were investigated may not represent the entire region or all LMIC. In addition, in presenting means, the data may differ from individual results, as substantial variations were identified. In this sense, it is relevant to pay attention to the data from each individual country to understand what these data reveal about the local reproductive health situation. Being currently pregnant was a filter for the DHS questions regarding reasons for not using contraceptives. However, it is important to understand whether these women had a contraceptive failure or whether they were not using any contraceptive methods (and what the reason was, should they have not been using any methods of contraception). Another limitation is that, by grouping reasons such as nonuse due to religious concerns and nonuse due to partner opposition similarly under “other opposed” (this strategy was adopted because very few women mentioned religious prohibition), it is impossible to detect whether religious constraints predominated in some countries over other persons’ opposition. As well, we cannot rule out the possibility of differences in individuals’ understandings and interpretations of the survey questions, and thus in the way the themes under investigation were reported.

Nevertheless, the present study also has many strengths, such as the use of recent data (2010–2017) for a large number of countries and the inclusion of information about both married and unmarried women. This provides a comprehensive overview of the reasons for nonuse of contraception, and is a step forward in relation to previous studies [9, 11]. In addition, the present study only focused on, for the calculation of the indicator, women in need of contraception and provided knowledge on reasons why contraceptive methods are not used, with stratification by subgroups. By directly pinpointing groups in need of interventions, we can better fulfill the contraceptive needs of women.

Understanding the reasons behind why women are not using contraceptives has many potential implications for reproductive health policies. Identifying the opinions of women themselves on the topic and detecting possible difficulties they face may serve as a tool to help professionals from diverse fields keep in mind the importance of increasing access to reproductive health services, empower women to make their own reproductive decisions, and provide necessary information and support to ensure that women are capable of overcoming barriers.

## Conclusions

“Health concerns” and “infrequent sex” were the most frequent reasons for nonuse of contraception in many LMIC, though the prevalence of each reason varied across different countries and subgroups of women. To decrease the prevalence of demand for contraception not satisfied, it is necessary to design, test and implement contextualized interventions. Generally, it is necessary to listen to women’s beliefs and opinions, provide women with more information on the importance of using contraceptives to avoid unwanted pregnancies and sexually transmitted infections, inform them of the contraceptive methods available and their side effects, and increase their access to a wide range of contraceptive methods.

## Supplementary information

**Additional file 1.** Reason respondent opposed according to stratifiers in women (15–49 years) with demand for contraception not satisfied.

**Additional file 2.** Reason other opposed according to stratifiers in women (15–49 years old) with demand for contraception not satisfied.

**Additional file 3.** Reason lack of knowledge according to stratifiers in women (15–49 years old) with demand for contraception not satisfied.

**Additional file 4.** Reason health concerns according to stratifiers in women (15–49 years old) with demand for contraception not satisfied.

**Additional file 5.** Reason reason lack of access according to stratifiers in women (15–49 years old) with demand for contraception not satisfied.

**Additional file 6.** Reason method related according to stratifiers in women (15–49 years old) with demand for contraception not satisfied.

**Additional file 7.** Reason fatalistic according to stratifiers in women (15–49 years old) with demand for contraception not satisfied.

**Additional file 8.** Reason infrequent sex according to stratifiers in women (15–49 years old) with demand for contraception not satisfied.

**Additional file 9.** Slope Index of Inequality (SII) calculated employing wealth index.

## Data Availability

The Demographic and Health Surveys (DHS) used in the present analyses are publicly available at < https://dhsprogram.com/data/available-datasets.cfm>.

## Abbreviations

CI: Confidence Interval
DHS: Demographic and Health Surveys
ISO: International Organization for Standardization
LMIC: Low and middle-income countries
MICS: Multiple Indicator Cluster Surveys
SII: Slope Index of Inequality
