# Supplementary material for: Reasons for nonuse of contraceptive methods by women with demand for contraception not satisfied: an assessment of low and middle-income countries using demographic and health surveys
Source: Reprod Health. 2019 Oct 11;16:148. doi: 10.1186/s12978-019-0805-7 (PMC6788119; doi:10.1186/s12978-019-0805-7)
Supplement: Supplementary file 2 — Additional file 2. Reason other opposed according to stratifiers in women (15–49 years old) with demand for contraception not satisfied. [file 12978_2019_805_MOESM2_ESM.docx]

| **Additional file 2 –** Reason **other opposed** according to stratifiers in women (15-49 years old) with demand for contraception not satisfied. | | | | | | | | | | | | | |  |  |  |  |  |  |
| --- | --- | --- | --- | --- | --- | --- | --- | --- | --- | --- | --- | --- | --- | --- | --- | --- | --- | --- | --- |
|  |  | Marital status | | Area of residence | | Woman's education | | | Wealth index | | | | | Parity | | | Woman's age | | |
| Country | Year | Married | Unmarried | Urban | Rural | None | Primary | Secondary or Higher | Q1 | Q2 | Q3 | Q4 | Q5 | 0 | 1-2 | 3+ | 15-19 | 20-34 | 35-49 |
| Angola | 2015 | 10.7 | 6.3 | 10.7 | 8.4 | 7.0 | 13.5 | 7.8 | 8.1 | 8.0 | 14.3 | 12.2 | 5.3 | 11.5 | 8.0 | 10.7 | 7.6 | 9.8 | 11.2 |
| Armenia | 2015 | 5.2 | Na | 5.1 | 5.5 | Na | Na | 5.2 | 7.8 | 4.9 | 2.2 | 7.3 | 4.4 | .b | 5.7 | 4.4 | .b | 7.8 | 2.9 |
| Benin | 2011 | 14.8 | 3.3 | 15.4 | 13.1 | 15.0 | 11.5 | 14.0 | 12.2 | 12.1 | 13.7 | 16.1 | 15.6 | 4.6 | 12.0 | 15.7 | 6.5 | 13.8 | 15.9 |
| Burkina Faso | 2010 | 18.4 | 7.3 | 14.3 | 19.2 | 19.4 | 14.2 | 6.9 | 22.5 | 18.9 | 16.7 | 17.5 | 15.7 | 6.5 | 17.7 | 19.0 | 13.6 | 18.9 | 18.3 |
| Burundi | 2016 | 27.5 | 4.0 | 22.2 | 27.6 | 27.2 | 26.5 | 30.5 | 23.2 | 24.5 | 29.5 | 28.7 | 31.2 | .b | 25.5 | 27.6 | .b | 25.5 | 29.1 |
| Cambodia | 2014 | 1.9 | .b | 0.8 | 2.1 | 0.4 | 2.0 | 2.5 | 2.5 | 1.8 | 1.8 | 2.2 | 0.7 | 14.6 | 2.9 | 0.3 | 8.1 | 3.1 | 0.2 |
| Cameroon | 2011 | 13.4 | 4.4 | 10.3 | 15.0 | 20.0 | 13.0 | 6.5 | 17.5 | 14.5 | 11.7 | 9.4 | 10.7 | 4.2 | 10.3 | 14.5 | 9.9 | 14.2 | 11.3 |
| Chad | 2014 | 15.0 | 4.9 | 19.0 | 13.2 | 16.3 | 12.9 | 10.4 | 15.8 | 13.3 | 14.6 | 9.9 | 19.1 | 14.6 | 11.2 | 16.2 | 9.3 | 14.6 | 17.1 |
| Colombia | 2015 | 5.6 | 2.9 | 4.1 | 6.1 | 4.5 | 8.6 | 3.5 | 6.7 | 7.5 | 2.9 | 0.9 | 4.2 | 5.5 | 3.0 | 7.2 | 5.7 | 4.7 | 4.0 |
| Comoros | 2012 | 14.8 | .b | 7.0 | 16.8 | 15.4 | 14.3 | 13.6 | 18.7 | 13.1 | 18.4 | 9.6 | 10.6 | 14.0 | 11.0 | 16.2 | 12.9 | 14.2 | 15.5 |
| Congo Brazzaville | 2011 | 10.4 | 3.8 | 10.4 | 7.6 | 14.7 | 9.9 | 8.6 | 6.5 | 11.6 | 10.6 | 8.3 | 10.6 | 6.3 | 8.8 | 10.5 | 1.7 | 12.1 | 7.3 |
| Congo Democratic Republic | 2013 | 18.2 | 8.3 | 19.3 | 16.5 | 17.2 | 17.6 | 17.3 | 15.8 | 16.4 | 17.5 | 18.4 | 19.4 | 9.8 | 15.8 | 18.5 | 13.4 | 16.7 | 20.1 |
| Côte d’Ivoire | 2011 | 14.6 | 9.7 | 13.9 | 14.0 | 14.7 | 12.7 | 12.8 | 15.0 | 12.0 | 14.6 | 15.4 | 12.3 | 12.0 | 12.9 | 14.8 | 9.8 | 14.6 | 14.0 |
| Dominican Republic | 2013 | 3.7 | 2.3 | 3.2 | 3.5 | 0 | 4.6 | 2.9 | 3.0 | 1.9 | 9.4 | 0 | 2.6 | 6.6 | 1.8 | 3.1 | 5.9 | 2.2 | 3.9 |
| Ethiopia | 2016 | 13.2 | 15.8 | 9.5 | 13.6 | 12.9 | 15.0 | 8.5 | 13.1 | 16.3 | 12.7 | 11.2 | 11.1 | 20.8 | 13.1 | 12.9 | 30.1 | 15.1 | 8.1 |
| Gabon | 2012 | 12.0 | 7.8 | 11.2 | 9.9 | 42.4 | 12.2 | 7.6 | 10.8 | 12.9 | 15.8 | 10.1 | 1.6 | 6.9 | 13.3 | 10.4 | 14.3 | 11.4 | 8.8 |
| Gambia | 2013 | 17.2 | .b | 17.6 | 16.8 | 18.9 | 13.2 | 15.2 | 10.6 | 17.7 | 25.5 | 17.0 | 13.9 | 0 | 16.9 | 17.9 | 14.1 | 18.7 | 14.7 |
| Ghana | 2014 | 8.1 | 3.9 | 5.5 | 9.9 | 12.2 | 10.0 | 4.7 | 13.4 | 9.6 | 6.4 | 6.6 | 2.4 | 6.1 | 8.2 | 7.6 | 10.6 | 7.3 | 7.9 |
| Guatemala | 2014 | 10.9 | 9.0 | 8.5 | 11.8 | 13.7 | 11.1 | 7.3 | 16.6 | 12.1 | 7.8 | 4.9 | 5.8 | 10.9 | 9.2 | 12.3 | 16.2 | 10.5 | 8.8 |
| Guinea | 2012 | 15.5 | .b | 15.5 | 15.5 | 17.0 | 11.2 | 10.1 | 15.6 | 15.1 | 14.1 | 17.8 | 14.3 | .b | 7.5 | 18.9 | 6.3 | 13.6 | 20.7 |
| Haiti | 2016 | 12.3 | 8.0 | 11.7 | 11.3 | 12.3 | 9.9 | 12.2 | 12.0 | 12.4 | 9.0 | 12.4 | 11.1 | 11.0 | 11.3 | 11.7 | 10.6 | 10.8 | 12.8 |
| Honduras | 2011 | 9.6 | 4.1 | 4.3 | 13.0 | 20.2 | 11.3 | 3.3 | 20.8 | 7.9 | 9.8 | 5.2 | 1.2 | 7.9 | 5.8 | 12.8 | 8.7 | 8.4 | 10.0 |
| India | 2015 | 19.7 | 10.4 | 19.0 | 20.0 | 20.1 | 18.8 | 19.7 | 20.6 | 19.2 | 19.9 | 19.2 | 19.4 | 23.7 | 19.2 | 19.7 | 21.4 | 20.2 | 17.7 |
| Indonesia | 2012 | 1.8 | .b | 1.8 | 1.7 | 5.4 | 1.5 | 1.7 | 2.2 | 1.7 | 2.1 | 1.6 | 1.5 | 1.8 | 1.8 | 1.7 | 1.9 | 2.9 | 1.2 |
| Kenya | 2014 | 10.4 | 0 | 8.4 | 9.9 | 23.6 | 8.0 | 6.0 | 14.1 | 9.0 | 7.8 | 5.9 | 7.7 | 5.2 | 9.2 | 9.7 | 9.9 | 9.9 | 8.6 |
| Kyrgyzstan | 2012 | 17.2 | .b | 10.4 | 20.4 | Na | Na | 17.1 | 26.8 | 14.4 | 16.1 | 17.5 | 11.4 | .b | 17.5 | 17.0 | .b | 17.3 | 17.1 |
| Lesotho | 2014 | 6.2 | 0 | 2.3 | 6.6 | 13.6 | 5.7 | 4.8 | 7.3 | 3.0 | 7.3 | 8.1 | 1.7 | 1.3 | 4.9 | 6.8 | 0.9 | 5.7 | 6.4 |
| Liberia | 2013 | 11.4 | 12.8 | 14.8 | 7.8 | 14.3 | 11.2 | 9.3 | 8.0 | 11.6 | 14.2 | 9.0 | 16.8 | 15.6 | 9.1 | 12.3 | 14.1 | 11.4 | 10.9 |
| Malawi | 2015 | 5.6 | 2.6 | 3.1 | 5.8 | 8.0 | 5.8 | 2.0 | 5.3 | 5.6 | 6.0 | 5.0 | 4.6 | 2.5 | 5.2 | 5.6 | 3.8 | 6.0 | 4.7 |
| Mali | 2012 | 26.2 | .b | 24.5 | 26.9 | 26.2 | 27.9 | 26.7 | 27.6 | 24.7 | 24.4 | 29.0 | 26.8 | .b | 23.3 | 27.1 | 28.5 | 27.8 | 23.5 |
| Mozambique | 2015 | 7.6 | 3.9 | 2.7 | 8.9 | 9.5 | 7.5 | 1.9 | 12.5 | 6.8 | 9.5 | 4.5 | 2.1 | 1.9 | 7.4 | 7.5 | 3.3 | 8.3 | 7.4 |
| Myanmar | 2015 | 1.3 | .b | 1.2 | 1.3 | 2.2 | 1.5 | 0.1 | 2.4 | 1.1 | 0.7 | 1.0 | 0.5 | 8.8 | 1.1 | 0.7 | 9.4 | 1.7 | 0.6 |
| Namibia | 2013 | 11.5 | 4.1 | 10.8 | 9.2 | 15.4 | 10.2 | 8.6 | 12.4 | 9.7 | 7.0 | 8.5 | 11.1 | 3.6 | 10.9 | 10.5 | 1.0 | 7.8 | 14.1 |
| Nepal | 2016 | 5.3 | Na | 5.8 | 4.6 | 7.7 | 5.6 | 3.7 | 4.6 | 4.2 | 7.0 | 5.8 | 4.8 | 8.7 | 4.6 | 5.8 | 6.2 | 5.4 | 4.6 |
| Niger | 2012 | 11.7 | Na | 17.3 | 10.7 | 11.4 | 14.9 | 10.5 | 12.2 | 11.5 | 10.3 | 9.9 | 14.8 | 10.9 | 13.1 | 11.3 | 13.0 | 11.2 | 12.4 |
| Nigeria | 2013 | 18.0 | 11.2 | 18.1 | 17.8 | 21.5 | 15.6 | 15.0 | 19.9 | 15.2 | 22.7 | 15.2 | 15.5 | 24.5 | 16.6 | 18.3 | 18.1 | 18.2 | 17.3 |
| Philippines | 2017 | 7.9 | 6.5 | 7.0 | 8.4 | 10.9 | 7.4 | 7.9 | 10.7 | 7.5 | 7.4 | 5.3 | 7.9 | 8.2 | 10.0 | 6.2 | 5.6 | 10.3 | 6.2 |
| Rwanda | 2014 | 6.1 | 1.2 | 8.4 | 5.2 | 3.0 | 6.2 | 7.8 | 3.0 | 7.9 | 6.0 | 4.6 | 7.0 | 0 | 10.3 | 4.0 | .b | 6.8 | 4.7 |
| Senegal | 2017 | 21.1 | .b | 19.3 | 21.8 | 23.4 | 15.3 | 17.1 | 24.0 | 19.4 | 21.0 | 23.6 | 15.7 | 19.5 | 22.5 | 20.5 | 22.6 | 22.3 | 19.2 |
| Sierra Leone | 2013 | 10.5 | 8.0 | 12.7 | 9.2 | 10.5 | 10.6 | 9.0 | 9.2 | 7.8 | 9.9 | 12.5 | 12.4 | 6.1 | 8.6 | 11.6 | 4.9 | 10.4 | 11.6 |
| Tajikistan | 2012 | 12.8 | .b | 15.7 | 12.0 | 0 | 11.7 | 13.1 | 14.8 | 10.0 | 13.1 | 10.2 | 16.5 | .b | 11.8 | 13.4 | 9.2 | 14.0 | 10.9 |
| Tanzania | 2015 | 17.5 | 3.5 | 8.9 | 19.1 | 20.1 | 15.8 | 11.1 | 25.7 | 14.8 | 18.9 | 9.6 | 8.6 | 4.1 | 15.9 | 16.8 | 17.4 | 17.0 | 14.5 |
| Timor-Leste | 2016 | 4.0 | .b | 4.5 | 3.7 | 2.3 | 3.1 | 5.1 | 5.5 | 2.5 | 2.9 | 6.2 | 3.0 | .b | 5.1 | 3.5 | .b | 5.1 | 2.7 |
| Togo | 2013 | 9.6 | 3.3 | 8.0 | 9.9 | 11.5 | 6.5 | 9.2 | 13.4 | 9.9 | 7.9 | 5.7 | 9.3 | 3.9 | 8.8 | 9.9 | 4.7 | 9.4 | 9.6 |
| Uganda | 2016 | 11.5 | 3.5 | 4.3 | 12.4 | 11.5 | 12.5 | 5.4 | 16.3 | 12.1 | 10.9 | 8.1 | 3.9 | 8.5 | 12.1 | 10.4 | 13.4 | 12.8 | 6.7 |
| Zambia | 2013 | 11.2 | 0 | 6.0 | 11.7 | 8.1 | 12.0 | 6.3 | 13.4 | 12.3 | 9.5 | 5.8 | 5.9 | 0 | 12.4 | 9.9 | 8.3 | 11.0 | 9.2 |
| Zimbabwe | 2015 | 14.7 | 7.4 | 12.5 | 14.7 | 0 | 18.1 | 12.3 | 19.4 | 9.4 | 11.1 | 11.5 | 17.8 | .b | 13.2 | 13.3 | 18.9 | 16.0 | 10.7 |
| *Na (Not available) = missing values | | | | | | |  |  |  |  |  |  |  |  |  |  |  |  |  |
| #.b = n <25 |  |  |  |  |  |  |  |  |  |  |  |  |  |  |  |  |  |  |  |
