# Supplementary material for: Reasons for nonuse of contraceptive methods by women with demand for contraception not satisfied: an assessment of low and middle-income countries using demographic and health surveys
Source: Reprod Health. 2019 Oct 11;16:148. doi: 10.1186/s12978-019-0805-7 (PMC6788119; doi:10.1186/s12978-019-0805-7)
Supplement: Supplementary file 4 — Additional file 4. Reason health concerns according to stratifiers in women (15–49 years old) with demand for contraception not satisfied. [file 12978_2019_805_MOESM4_ESM.docx]

| **Additional file 4 –** Reason **health concerns** according to stratifiers in women (15-49 years old) with demand for contraception not satisfied. | | | | | | | | | | | | | | | | | | | |
| --- | --- | --- | --- | --- | --- | --- | --- | --- | --- | --- | --- | --- | --- | --- | --- | --- | --- | --- | --- |
|  |  | Marital status | | Area of residence | | Woman's education | | | Wealth index | | | | | Parity | | | Woman's age | | |
| Country | Year | Married | Unmarried | Urban | Rural | None | Primary | Secondary or Higher | Q1 | Q2 | Q3 | Q4 | Q5 | 0 | 1-2 | 3+ | 15-19 | 20-34 | 35-49 |
| Angola | 2015 | 13.7 | 11.2 | 16.2 | 7.2 | 7.7 | 16.1 | 14.0 | 5.3 | 6.0 | 13.9 | 20.7 | 19.0 | 13.5 | 11.4 | 14.1 | 6.3 | 12.0 | 18.5 |
| Armenia | 2015 | 15.0 | Na | 14.3 | 16.1 | Na | Na | 15.0 | 14.1 | 11.7 | 21.9 | 14.5 | 13.0 | .b | 16.8 | 11.1 | .b | 15.6 | 14.5 |
| Benin | 2011 | 19.9 | 17.5 | 24.4 | 15.8 | 17.8 | 21.8 | 26.2 | 12.0 | 14.7 | 14.0 | 25.3 | 28.1 | 16.7 | 17.7 | 20.8 | 7.1 | 19.1 | 22.7 |
| Burkina Faso | 2010 | 14.6 | 19.3 | 28.8 | 11.6 | 13.3 | 20.5 | 26.8 | 12.1 | 12.5 | 9.4 | 16.5 | 26.1 | 16.9 | 12.4 | 15.7 | 11.6 | 13.5 | 17.5 |
| Burundi | 2016 | 33.0 | 30.8 | 39.0 | 32.5 | 33.3 | 34.0 | 25.7 | 29.9 | 35.8 | 32.1 | 36.2 | 30.4 | .b | 30.4 | 33.7 | .b | 33.8 | 32.6 |
| Cambodia | 2014 | 36.3 | .b | 30.9 | 36.8 | 39.6 | 36.9 | 32.1 | 42.7 | 38.9 | 35.6 | 31.3 | 27.6 | 42.5 | 34.1 | 37.5 | 52.6 | 33.6 | 37.5 |
| Cameroon | 2011 | 21.9 | 21.9 | 27.9 | 16.9 | 17.8 | 22.3 | 25.1 | 10.3 | 20.0 | 26.4 | 27.7 | 26.3 | 17.1 | 16.7 | 24.4 | 15.3 | 19.9 | 27.7 |
| Chad | 2014 | 7.8 | 8.5 | 13.3 | 5.9 | 6.5 | 9.9 | 10.0 | 4.6 | 6.1 | 8.3 | 4.2 | 14.5 | 5.5 | 5.4 | 8.9 | 3.6 | 6.2 | 13.5 |
| Colombia | 2015 | 20.8 | 11.7 | 17.1 | 18.9 | 18.8 | 17.9 | 17.4 | 20.6 | 17.5 | 17.2 | 17.1 | 13.6 | 15.8 | 18.6 | 17.6 | 12.1 | 20.6 | 15.6 |
| Comoros | 2012 | 29.6 | .b | 32.8 | 28.3 | 24.2 | 42.6 | 27.9 | 20.7 | 29.4 | 36.8 | 29.1 | 33.3 | 10.0 | 22.6 | 33.8 | 20.0 | 26.0 | 37.7 |
| Congo Brazzaville | 2011 | 18.2 | 9.7 | 17.9 | 15.3 | 14.8 | 11.3 | 19.9 | 12.6 | 12.8 | 23.7 | 16.0 | 21.8 | 4.9 | 15.4 | 20.2 | 8.5 | 16.2 | 24.7 |
| Congo Democratic Republic | 2013 | 17.7 | 26.4 | 21.4 | 17.0 | 19.8 | 16.8 | 19.5 | 14.3 | 16.3 | 18.8 | 19.6 | 24.4 | 9.7 | 18.1 | 18.9 | 8.7 | 18.5 | 20.9 |
| Côte d’Ivoire | 2011 | 22.8 | 34.3 | 31.1 | 19.3 | 24.1 | 23.4 | 26.4 | 17.7 | 21.1 | 23.0 | 30.9 | 29.4 | 37.8 | 21.5 | 24.0 | 18.9 | 24.9 | 24.6 |
| Dominican Republic | 2013 | 31.1 | 22.8 | 30.2 | 22.9 | 19.1 | 31.0 | 28.3 | 22.0 | 36.5 | 29.5 | 24.9 | 32.4 | 23.6 | 30.9 | 29.3 | 19.6 | 32.2 | 28.2 |
| Ethiopia | 2016 | 16.5 | 9.0 | 18.9 | 16.1 | 15.0 | 17.7 | 26.6 | 11.8 | 17.3 | 15.5 | 16.7 | 23.6 | 9.7 | 9.3 | 18.6 | 9.3 | 11.0 | 25.0 |
| Gabon | 2012 | 17.8 | 16.1 | 18.5 | 11.3 | 11.1 | 15.2 | 18.9 | 12.5 | 17.3 | 17.0 | 22.9 | 17.0 | 13.4 | 15.4 | 19.5 | 7.9 | 17.4 | 21.3 |
| Gambia | 2013 | 12.6 | .b | 14.5 | 10.9 | 12.3 | 13.3 | 13.3 | 11.4 | 9.1 | 14.6 | 11.5 | 17.2 | 17.0 | 11.7 | 13.0 | 13.1 | 10.9 | 16.1 |
| Ghana | 2014 | 47.1 | 44.5 | 49.9 | 43.8 | 35.1 | 56.5 | 49.1 | 32.4 | 47.1 | 48.2 | 52.9 | 54.3 | 37.3 | 43.6 | 49.4 | 39.4 | 44.0 | 51.2 |
| Guatemala | 2014 | 17.1 | 14.4 | 19.6 | 15.8 | 18.1 | 15.9 | 18.3 | 16.4 | 15.7 | 12.8 | 20.4 | 22.6 | 14.7 | 14.5 | 19.7 | 7.3 | 16.8 | 22.1 |
| Guinea | 2012 | 10.9 | .b | 17.5 | 8.3 | 10.4 | 7.6 | 18.5 | 6.1 | 5.6 | 9.9 | 14.7 | 18.4 | .b | 6.6 | 12.4 | 3.6 | 8.8 | 16.1 |
| Haiti | 2016 | 43.0 | 28.6 | 41.6 | 39.2 | 43.6 | 41.1 | 38.1 | 36.9 | 36.2 | 39.3 | 44.5 | 42.7 | 24.4 | 40.6 | 46.5 | 18.0 | 37.5 | 51.0 |
| Honduras | 2011 | 20.2 | 13.2 | 19.9 | 19.3 | 25.2 | 20.7 | 16.7 | 19.2 | 21.9 | 20.3 | 19.2 | 17.4 | 22.5 | 15.5 | 23.2 | 11.1 | 17.5 | 25.2 |
| India | 2015 | 7.4 | 0 | 9.5 | 6.4 | 5.4 | 6.4 | 8.5 | 5.0 | 6.4 | 8.0 | 9.0 | 9.0 | 5.6 | 7.7 | 7.1 | 4.9 | 6.9 | 9.5 |
| Indonesia | 2012 | 29.9 | .b | 32.6 | 26.7 | 15.8 | 24.6 | 34.9 | 25.9 | 26.0 | 31.1 | 30.1 | 34.7 | 7.9 | 30.8 | 29.1 | 18.8 | 32.4 | 28.6 |
| Kenya | 2014 | 43.4 | 28.1 | 44.6 | 40.7 | 23.5 | 40.7 | 52.7 | 32.3 | 46.1 | 43.0 | 48.5 | 44.0 | 34.2 | 42.6 | 42.2 | 23.8 | 40.5 | 46.1 |
| Kyrgyzstan | 2012 | 20.9 | .b | 30.2 | 16.4 | Na | Na | 20.9 | 15.9 | 22.5 | 13.4 | 20.6 | 32.6 | .b | 15.1 | 25.9 | .b | 15.8 | 28.1 |
| Lesotho | 2014 | 12.1 | 2.8 | 11.7 | 10.8 | 7.6 | 14.7 | 6.7 | 12.7 | 9.2 | 13.2 | 9.7 | 10.7 | 1.3 | 10.0 | 13.9 | 3.4 | 10.7 | 13.7 |
| Liberia | 2013 | 25.2 | 34.4 | 31.4 | 22.2 | 22.4 | 21.7 | 37.6 | 19.1 | 23.8 | 26.1 | 31.4 | 37.3 | 28.7 | 25.2 | 28.4 | 24.8 | 25.9 | 31.5 |
| Malawi | 2015 | 22.5 | 3.1 | 31.4 | 17.7 | 15.6 | 20.3 | 23.3 | 14.7 | 16.9 | 18.8 | 22.6 | 27.2 | 4.4 | 17.8 | 23.5 | 4.7 | 20.5 | 24.6 |
| Mali | 2012 | 9.3 | .b | 13.9 | 8.2 | 9.3 | 10.7 | 6.5 | 6.0 | 7.8 | 7.7 | 11.3 | 14.3 | .b | 5.2 | 10.8 | 7.2 | 7.8 | 12.3 |
| Mozambique | 2015 | 10.0 | 7.0 | 10.0 | 9.5 | 9.5 | 9.3 | 10.8 | 8.4 | 10.1 | 8.6 | 6.0 | 15.4 | 2.7 | 6.7 | 12.1 | 2.1 | 8.3 | 16.1 |
| Myanmar | 2015 | 32.7 | .b | 31.2 | 33.0 | 32.6 | 33.4 | 31.4 | 35.9 | 32.9 | 36.4 | 29.8 | 25.6 | 27.9 | 31.0 | 34.6 | 13.0 | 34.1 | 32.5 |
| Namibia | 2013 | 27.2 | 22.9 | 30.3 | 23.4 | 10.8 | 25.7 | 29.8 | 19.6 | 26.1 | 26.2 | 41.7 | 17.4 | 7.9 | 30.9 | 27.0 | 4.8 | 28.2 | 28.9 |
| Nepal | 2016 | 15.0 | Na | 17.3 | 11.7 | 17.5 | 16.1 | 13.0 | 14.8 | 13.1 | 11.3 | 13.1 | 22.6 | 2.6 | 14.2 | 19.5 | 2.8 | 12.8 | 25.4 |
| Niger | 2012 | 6.2 | Na | 15.3 | 4.5 | 5.2 | 8.0 | 18.4 | 3.2 | 4.5 | 5.8 | 5.4 | 12.4 | 3.4 | 5.6 | 6.5 | 6.0 | 4.9 | 9.1 |
| Nigeria | 2013 | 19.5 | 20.8 | 26.8 | 15.8 | 13.5 | 22.5 | 24.9 | 9.7 | 13.0 | 21.7 | 27.4 | 26.6 | 9.3 | 14.4 | 22.0 | 6.4 | 17.2 | 25.5 |
| Philippines | 2017 | 26.8 | 16.8 | 27.4 | 25.6 | 20.5 | 28.2 | 26.0 | 26.0 | 28.0 | 24.1 | 25.3 | 27.9 | 19.1 | 27.6 | 26.0 | 17.7 | 27.9 | 25.9 |
| Rwanda | 2014 | 34.6 | 30.9 | 38.5 | 33.5 | 40.2 | 33.0 | 30.9 | 27.8 | 36.4 | 35.2 | 37.1 | 36.1 | 7.1 | 35.8 | 34.6 | .b | 30.9 | 40.1 |
| Senegal | 2017 | 15.1 | .b | 15.7 | 14.8 | 15.3 | 17.9 | 10.9 | 12.1 | 15.1 | 17.5 | 18.4 | 13.0 | 9.7 | 9.5 | 17.9 | 6.4 | 11.9 | 20.9 |
| Sierra Leone | 2013 | 11.6 | 29.7 | 17.6 | 11.6 | 11.8 | 14.5 | 18.2 | 11.7 | 10.2 | 12.5 | 16.8 | 16.9 | 22.1 | 11.7 | 13.4 | 13.9 | 12.8 | 14.2 |
| Tajikistan | 2012 | 14.5 | .b | 13.3 | 14.8 | 13.5 | 12.9 | 14.6 | 12.5 | 14.6 | 15.9 | 15.9 | 13.7 | .b | 11.4 | 16.8 | 14.1 | 12.3 | 18.5 |
| Tanzania | 2015 | 40.0 | 43.0 | 48.3 | 37.0 | 35.7 | 41.0 | 44.3 | 32.0 | 40.5 | 38.7 | 41.5 | 51.2 | 30.2 | 32.3 | 44.7 | 28.4 | 38.6 | 45.7 |
| Timor-Leste | 2016 | 35.7 | .b | 35.0 | 36.2 | 34.0 | 37.9 | 35.9 | 38.4 | 35.4 | 34.2 | 37.2 | 34.1 | .b | 31.0 | 39.0 | .b | 33.5 | 40.0 |
| Togo | 2013 | 34.5 | 32.5 | 38.8 | 31.5 | 36.6 | 31.7 | 34.6 | 26.2 | 36.9 | 32.9 | 39.4 | 36.2 | 27.8 | 29.6 | 38.2 | 17.4 | 32.9 | 40.8 |
| Uganda | 2016 | 22.0 | 13.8 | 24.0 | 20.6 | 20.8 | 21.5 | 20.9 | 19.5 | 20.9 | 20.8 | 21.2 | 25.2 | 19.2 | 17.4 | 23.0 | 16.2 | 20.1 | 24.9 |
| Zambia | 2013 | 31.8 | 16.4 | 41.2 | 25.2 | 34.8 | 27.6 | 33.7 | 21.8 | 25.4 | 27.8 | 37.3 | 49.4 | 11.1 | 24.5 | 34.1 | 9.1 | 28.2 | 39.2 |
| Zimbabwe | 2015 | 19.6 | 12.6 | 26.5 | 15.4 | 8.0 | 14.2 | 22.5 | 10.4 | 15.2 | 18.9 | 30.9 | 20.7 | .b | 18.8 | 20.2 | 0 | 16.9 | 25.1 |
| *Na (Not available) = missing values | | | | | | |  |  |  |  |  |  |  |  |  |  |  |  |  |
| #.b = n <25 |  |  |  |  |  |  |  |  |  |  |  |  |  |  |  |  |  |  |  |
