# Supplementary material for: Reasons for nonuse of contraceptive methods by women with demand for contraception not satisfied: an assessment of low and middle-income countries using demographic and health surveys
Source: Reprod Health. 2019 Oct 11;16:148. doi: 10.1186/s12978-019-0805-7 (PMC6788119; doi:10.1186/s12978-019-0805-7)
Supplement: Supplementary file 6 — Additional file 6. Reason method related according to stratifiers in women (15–49 years old) with demand for contraception not satisfied. [file 12978_2019_805_MOESM6_ESM.docx]

| **Additional file 6 –** Reason **method related** according to stratifiers in women (15-49 years old) with demand for contraception not satisfied. | | | | | | | | | | | | | | | | | | | |
| --- | --- | --- | --- | --- | --- | --- | --- | --- | --- | --- | --- | --- | --- | --- | --- | --- | --- | --- | --- |
|  |  | Marital status | | Area of residence | | Woman's education | | | Wealth index | | | | | Parity | | | Woman's age | | |
| Country | Year | Married | Unmarried | Urban | Rural | None | Primary | Secondary or Higher | Q1 | Q2 | Q3 | Q4 | Q5 | 0 | 1-2 | 3+ | 15-19 | 20-34 | 35-49 |
| Angola | 2015 | 2.1 | 0.2 | 1.5 | 2.3 | 1.8 | 1.8 | 1.8 | 2.7 | 2.0 | 1.2 | 0.7 | 2.9 | 0.6 | 2.1 | 1.8 | 1.2 | 2.0 | 1.7 |
| Armenia | 2015 | 2.1 | Na | 2.8 | 1.1 | Na | Na | 2.1 | 0 | 2.2 | 3.2 | 4.3 | 1.0 | .b | 1.8 | 2.8 | .b | 0.5 | 3.7 |
| Benin | 2011 | 2.7 | 1.2 | 3.1 | 2.2 | 1.9 | 5.1 | 2.7 | 1.8 | 1.6 | 2.3 | 2.8 | 3.9 | 1.5 | 1.5 | 3.1 | 1.7 | 2.2 | 3.5 |
| Burkina Faso | 2010 | 0.8 | 0 | 1.1 | 0.7 | 0.8 | 1.0 | 0.4 | 0.2 | 1.3 | 0.7 | 0.8 | 0.9 | 0 | 0.5 | 0.9 | 0.5 | 0.6 | 1.2 |
| Burundi | 2016 | 0.8 | 4.9 | 1.0 | 0.8 | 1.0 | 0.5 | 1.8 | 1.1 | 0.4 | 0.4 | 1.1 | 1.4 | .b | 1.1 | 0.8 | .b | 0.5 | 1.2 |
| Cambodia | 2014 | 2.1 | .b | 0.4 | 2.4 | 4.8 | 1.4 | 2.0 | 2.4 | 1.9 | 2.3 | 1.7 | 2.1 | 0 | 2.5 | 2.0 | 1.4 | 1.7 | 2.6 |
| Cameroon | 2011 | 2.5 | 2.5 | 3.1 | 2.0 | 2.3 | 2.2 | 3.1 | 2.5 | 1.8 | 1.6 | 4.5 | 2.1 | 4.1 | 2.5 | 2.4 | 1.7 | 2.3 | 3.1 |
| Chad | 2014 | 0.4 | 0 | 1.1 | 0.1 | 0.4 | 0.4 | 0.4 | 0 | 0.3 | 0 | 0.4 | 1.1 | 0 | 0.5 | 0.3 | 0.5 | 0.4 | 0.4 |
| Colombia | 2015 | Na | Na | Na | Na | Na | Na | Na | Na | Na | Na | Na | Na | Na | Na | Na | Na | Na | Na |
| Comoros | 2012 | 1.3 | .b | 0.8 | 1.4 | 1.7 | 0.4 | 1.3 | 2.2 | 1.6 | 1.2 | 0.5 | 0.5 | 3.4 | 1.3 | 1.1 | 2.0 | 1.7 | 0.3 |
| Congo Brazzaville | 2011 | 1.2 | 2.3 | 1.6 | 0.9 | 0 | 1.8 | 1.3 | 0 | 2.2 | 1.1 | 2.6 | 0 | 4.0 | 0.8 | 1.4 | 2.1 | 0.8 | 2.4 |
| Congo Democratic Republic | 2013 | 1.4 | 3.9 | 2.0 | 1.3 | 0.6 | 1.2 | 2.4 | 1.3 | 1.0 | 1.0 | 2.4 | 2.2 | 4.9 | 1.8 | 1.3 | 3.5 | 1.4 | 1.2 |
| Côte d’Ivoire | 2011 | 2.1 | 0.2 | 1.0 | 2.4 | 2.2 | 1.9 | 0.3 | 0.5 | 3.5 | 3.4 | 1.1 | 1.0 | 1.5 | 1.4 | 2.2 | 0 | 2.2 | 1.7 |
| Dominican Republic | 2013 | 1.3 | 1.0 | 1.4 | 0.3 | 1.4 | 1.5 | 1.1 | 1.1 | 0.4 | 3.4 | 1.0 | 0 | 0.8 | 1.0 | 2.2 | 2.2 | 1.4 | 0 |
| Ethiopia | 2016 | 1.7 | 0 | 1.3 | 1.7 | 1.8 | 1.7 | 0.1 | 1.0 | 1.8 | 1.9 | 2.9 | 0.8 | 1.6 | 0.2 | 2.1 | 0 | 1.9 | 1.7 |
| Gabon | 2012 | 5.1 | 3.4 | 4.7 | 4.7 | 0.5 | 2.1 | 6.1 | 2.9 | 4.7 | 2.0 | 10.1 | 3.9 | 4.4 | 4.6 | 4.8 | 3.1 | 4.7 | 5.3 |
| Gambia | 2013 | 0.5 | .b | 0.4 | 0.5 | 0.1 | 0.9 | 1.1 | 0 | 0.4 | 1.2 | 0.6 | 0 | 0 | 0.6 | 0.4 | 0 | 0.3 | 0.8 |
| Ghana | 2014 | 5.4 | 5.4 | 5.9 | 4.8 | 5.8 | 8.8 | 3.9 | 4.1 | 4.3 | 6.2 | 6.1 | 6.2 | 7.4 | 3.1 | 6.3 | 8.9 | 3.7 | 6.9 |
| Guatemala | 2014 | 0.8 | 0.7 | 1.3 | 0.6 | 1.0 | 0.5 | 1.4 | 0.5 | 0.2 | 1.4 | 1.0 | 1.2 | 1.0 | 0.8 | 0.8 | 0.8 | 0.8 | 0.8 |
| Guinea | 2012 | 0.3 | .b | 0.9 | 0.1 | 0.3 | 0.9 | 0 | 0.4 | 0 | 0 | 0.1 | 1.2 | .b | 0.2 | 0.4 | 0.7 | 0.3 | 0.3 |
| Haiti | 2016 | 1.6 | 3.4 | 1.8 | 2.0 | 2.2 | 1.6 | 2.1 | 2.0 | 2.3 | 1.8 | 1.8 | 2.0 | 3.8 | 1.3 | 1.9 | 4.1 | 1.7 | 1.8 |
| Honduras | 2011 | 0.6 | 0 | 0.2 | 0.8 | 0.9 | 0.6 | 0.3 | 1.1 | 0 | 1.1 | 0.4 | 0 | 0 | 0.4 | 0.8 | 0 | 0.4 | 0.8 |
| India | 2015 | 1.0 | 0 | 1.1 | 0.9 | 0.6 | 0.6 | 1.2 | 0.5 | 0.9 | 1.1 | 1.4 | 1.0 | 0.6 | 1.1 | 0.8 | 0.4 | 1.0 | 0.9 |
| Indonesia | 2012 | 4.1 | .b | 5.6 | 2.3 | 3.5 | 3.1 | 4.8 | 1.9 | 2.4 | 1.4 | 6.0 | 7.4 | 1.7 | 3.6 | 4.7 | 0 | 1.7 | 5.3 |
| Kenya | 2014 | 1.0 | 0 | 0.4 | 1.2 | 0.3 | 1.0 | 1.0 | 1.0 | 1.1 | 1.5 | 0.6 | 0.4 | 1.9 | 0.4 | 1.1 | 0 | 0.5 | 1.6 |
| Kyrgyzstan | 2012 | 0.2 | .b | 1.6 | 0.3 | Na | Na | 0.8 | 0.8 | 0 | 0.5 | 0 | 2.9 | .b | 0.2 | 0.3 | .b | 0.1 | 1.6 |
| Lesotho | 2014 | 1.9 | 0 | 0.9 | 2.0 | 0 | 2.1 | 1.2 | 0 | 4.9 | 1.4 | 1.4 | 0.7 | 0 | 2.2 | 1.3 | 0 | 1.6 | 2.3 |
| Liberia | 2013 | 4.5 | 11.1 | 5.7 | 6.3 | 6.0 | 6.9 | 5.2 | 4.7 | 9.9 | 4.1 | 5.2 | 6.4 | 9.6 | 6.2 | 4.9 | 6.9 | 5.8 | 5.9 |
| Malawi | 2015 | 2.4 | 0.1 | 3.3 | 1.9 | 2.3 | 1.7 | 3.2 | 2.9 | 1.6 | 1.5 | 1.8 | 2.8 | 0.1 | 2.5 | 2.2 | 1.4 | 2.3 | 2.2 |
| Mali | 2012 | 0.8 | .b | 0.3 | 0.9 | 0.9 | 0.8 | 0 | 0 | 1.1 | 1.9 | 0.2 | 0.6 | .b | 0.5 | 0.9 | 1.1 | 0.9 | 0.6 |
| Mozambique | 2015 | 2.5 | 1.9 | 4.8 | 1.5 | 1.8 | 1.8 | 5.3 | 2.0 | 1.4 | 1.9 | 3.3 | 3.7 | 3.3 | 2.8 | 2.1 | 0.8 | 3.7 | 1.2 |
| Myanmar | 2015 | 2.7 | .b | 3.2 | 2.6 | 2.5 | 3.1 | 2.3 | 4.1 | 1.5 | 2.0 | 2.6 | 3.2 | 7.1 | 2.9 | 2.1 | 13.8 | 3.5 | 1.8 |
| Namibia | 2013 | 1.0 | 1.1 | 0.6 | 1.3 | 0.8 | 0.7 | 1.2 | 0.4 | 1.8 | 1.5 | 1.4 | 0 | 1.1 | 0.6 | 1.3 | 1.3 | 0.8 | 1.2 |
| Nepal | 2016 | 1.2 | Na | 2.0 | 0.1 | 1.1 | 0 | 1.8 | 0.9 | 0.1 | 0 | 0 | 5.3 | 0 | 1.6 | 0.9 | 0 | 1.3 | 1.5 |
| Niger | 2012 | 4.1 | Na | 2.5 | 4.4 | 4.4 | 2.5 | 3.6 | 3.8 | 4.9 | 3.9 | 6.1 | 2.0 | 0 | 2.9 | 4.7 | 2.9 | 3.9 | 4.9 |
| Nigeria | 2013 | 2.9 | 4.2 | 2.9 | 3.0 | 2.0 | 2.9 | 4.1 | 2.6 | 2.3 | 2.0 | 3.4 | 4.8 | 3.3 | 2.7 | 3.0 | 2.0 | 2.4 | 4.0 |
| Philippines | 2017 | 0.3 | 0 | 0.1 | 0.5 | 0 | 0.9 | 0.2 | 0.6 | 0.9 | 0 | 0 | 0 | 0 | 0.5 | 0.2 | 0 | 0.6 | 0.1 |
| Rwanda | 2014 | 2.3 | 2.3 | 2.7 | 2.2 | 2.4 | 2.4 | 1.6 | 2.1 | 1.6 | 2.2 | 3.5 | 2.2 | 0 | 3.5 | 1.8 | .b | 2.2 | 2.5 |
| Senegal | 2017 | 0.3 | .b | 0.1 | 0.4 | 0.3 | 0.6 | 0 | 0.3 | 0.4 | 0.5 | 0.3 | 0 | 0 | 0.2 | 0.4 | 0.9 | 0.4 | 0.1 |
| Sierra Leone | 2013 | 2.1 | 3.0 | 2.9 | 1.9 | 1.7 | 2.8 | 3.4 | 2.0 | 2.1 | 1.1 | 2.7 | 3.2 | 4.4 | 2.3 | 1.9 | 1.9 | 2.0 | 2.6 |
| Tajikistan | 2012 | 1.0 | .b | 0.2 | 1.2 | 0 | 0 | 1.1 | 1.4 | 0.5 | 1.4 | 1.2 | 0.2 | .b | 0.6 | 1.3 | 0 | 1.2 | 0.8 |
| Tanzania | 2015 | 1.2 | 2.3 | 3.0 | 0.6 | 0.6 | 1.0 | 3.4 | 1.5 | 0.8 | 0.4 | 2.5 | 1.2 | 0 | 1.4 | 1.3 | 1.7 | 1.4 | 1.1 |
| Timor-Leste | 2016 | 2.4 | .b | 1.7 | 2.7 | 3.6 | 0.6 | 2.4 | 2.5 | 4.3 | 2.2 | 1.4 | 1.7 | .b | 4.8 | 1.1 | .b | 3.3 | 1.3 |
| Togo | 2013 | 2.8 | 1.8 | 2.3 | 2.9 | 3.3 | 1.7 | 3.1 | 6.2 | 1.3 | 1.1 | 2.7 | 2.1 | 1.7 | 1.9 | 3.3 | 1.1 | 2.9 | 2.7 |
| Uganda | 2016 | 0.9 | 0.9 | 1.4 | 0.8 | 2.2 | 0.5 | 1.5 | 0.5 | 0.6 | 1.5 | 1.1 | 1.2 | 0 | 1.3 | 0.8 | 0.4 | 0.7 | 1.5 |
| Zambia | 2013 | 1.2 | 1.2 | 1.1 | 1.2 | 0.6 | 1.3 | 1.2 | 0.8 | 2.0 | 0.5 | 0.6 | 2.4 | 0 | 1.1 | 1.3 | 0.2 | 1.2 | 1.4 |
| Zimbabwe | 2015 | 1.6 | 2.3 | 1.1 | 1.9 | 0 | 1.6 | 1.8 | 1.2 | 3.9 | 0.5 | 1.2 | 1.3 | .b | 1.5 | 1.5 | 2.4 | 1.4 | 1.7 |
| *Na (Not available) = missing values | | | |  |  |  |  |  |  |  |  |  |  |  |  |  |  |  |  |
| #.b = n <25 |  |  |  |  |  |  |  |  |  |  |  |  |  |  |  |  |  |  |  |
