# Supplementary material for: Reasons for nonuse of contraceptive methods by women with demand for contraception not satisfied: an assessment of low and middle-income countries using demographic and health surveys
Source: Reprod Health. 2019 Oct 11;16:148. doi: 10.1186/s12978-019-0805-7 (PMC6788119; doi:10.1186/s12978-019-0805-7)
Supplement: Supplementary file 8 — Additional file 8. Reason infrequent sex according to stratifiers in women (15–49 years old) with demand for contraception not satisfied. [file 12978_2019_805_MOESM8_ESM.docx]

| **Additional file 8 –** Reason **infrequent sex** according to stratifiers in women (15-49 years old) with demand for contraception not satisfied. | | | | | | | | | | | | | | | | | | | |
| --- | --- | --- | --- | --- | --- | --- | --- | --- | --- | --- | --- | --- | --- | --- | --- | --- | --- | --- | --- |
|  |  | Marital status | | Area of residence | | Woman's education | | | Wealth index | | | | | Parity | | | Woman's age | | |
| Country | Year | Married | Unmarried | Urban | Rural | None | Primary | Secondary or Higher | Q1 | Q2 | Q3 | Q4 | Q5 | 0 | 1-2 | 3+ | 15-19 | 20-34 | 35-49 |
| Angola | 2015 | 5.4 | 14.6 | 7.3 | 5.7 | 6.3 | 4.9 | 9.6 | 4.5 | 8.3 | 6.2 | 7.0 | 7.7 | 13.3 | 9.5 | 5.1 | 8.9 | 6.9 | 5.9 |
| Armenia | 2015 | 25.8 | Na | 22.8 | 30.9 | Na | Na | 25.8 | 38.2 | 30.2 | 24.2 | 26.5 | 14.6 | .b | 22.3 | 33.9 | .b | 23.4 | 28.2 |
| Benin | 2011 | 15.8 | 16.2 | 10.4 | 20.4 | 16.4 | 13.8 | 15.5 | 17.1 | 17.4 | 19.5 | 16.6 | 10.0 | 15.6 | 19.5 | 14.2 | 18.8 | 16.6 | 13.9 |
| Burkina Faso | 2010 | 22.7 | 34.6 | 26.3 | 22.0 | 21.4 | 26.5 | 39.6 | 20.1 | 16.8 | 25.1 | 27.3 | 24.8 | 37.0 | 21.9 | 22.7 | 23.5 | 20.6 | 26.5 |
| Burundi | 2016 | 11.3 | 40.9 | 13.2 | 11.6 | 9.5 | 13.2 | 19.5 | 14.2 | 12.2 | 8.5 | 11.1 | 12.4 | .b | 15.3 | 10.7 | .b | 12.2 | 11.0 |
| Cambodia | 2014 | 45.3 | .b | 61.3 | 43.3 | 45.5 | 42.5 | 51.9 | 36.2 | 41.7 | 48.4 | 48.2 | 58.5 | 34.8 | 44.3 | 47.1 | 26.1 | 40.6 | 51.9 |
| Cameroon | 2011 | 28.7 | 38.1 | 32.3 | 26.7 | 15.3 | 31.3 | 38.5 | 15.6 | 32.5 | 34.9 | 30.5 | 33.8 | 49.6 | 33.9 | 26.0 | 37.6 | 28.7 | 27.5 |
| Chad | 2014 | 17.5 | 35.9 | 17.1 | 18.2 | 17.0 | 19.3 | 20.0 | 18.5 | 15.1 | 20.6 | 20.1 | 16.2 | 37.0 | 19.0 | 16.9 | 24.9 | 15.8 | 20.6 |
| Colombia | 2015 | 19.8 | 39.1 | 28.5 | 21.3 | 17.4 | 21.6 | 28.5 | 17.6 | 25.4 | 31.3 | 30.0 | 32.6 | 34.1 | 25.4 | 19.2 | 29.2 | 29.5 | 21.8 |
| Comoros | 2012 | 11.0 | .b | 17.7 | 9.2 | 7.2 | 10.6 | 18.0 | 8.1 | 8.9 | 9.7 | 11.5 | 20.5 | 33.0 | 14.6 | 8.1 | 15.6 | 12.0 | 8.6 |
| Congo Brazzaville | 2011 | 13.7 | 24.3 | 13.6 | 18.1 | 13.5 | 11.5 | 17.0 | 17.7 | 13.6 | 16.0 | 14.4 | 14.6 | 13.4 | 14.0 | 16.3 | 18.0 | 14.2 | 15.9 |
| Congo Democratic Republic | 2013 | 19.0 | 26.8 | 21.2 | 18.9 | 16.3 | 19.6 | 21.2 | 19.8 | 19.4 | 15.9 | 21.8 | 21.7 | 20.7 | 19.2 | 19.7 | 16.8 | 18.4 | 23.0 |
| Côte d’Ivoire | 2011 | 15.6 | 11.8 | 16.8 | 13.9 | 12.3 | 17.1 | 25.4 | 14.1 | 13.4 | 12.6 | 16.6 | 19.7 | 9.7 | 18.8 | 13.7 | 14.1 | 13.8 | 18.5 |
| Dominican Republic | 2013 | 21.2 | 31.4 | 23.6 | 25.9 | 26.1 | 16.5 | 27.2 | 18.4 | 23.8 | 22.5 | 31.6 | 22.2 | 27.3 | 23.1 | 22.6 | 22.2 | 26.6 | 19.5 |
| Ethiopia | 2016 | 8.7 | 22.5 | 21.3 | 7.9 | 8.4 | 7.2 | 27.4 | 8.0 | 6.3 | 10.0 | 7.0 | 17.7 | 21.8 | 14.5 | 6.8 | 12.4 | 8.8 | 8.8 |
| Gabon | 2012 | 17.5 | 17.1 | 17.2 | 18.6 | 16.6 | 12.3 | 19.5 | 13.6 | 18.5 | 10.8 | 21.0 | 26.2 | 21.3 | 17.3 | 16.6 | 20.0 | 17.4 | 16.4 |
| Gambia | 2013 | 14.8 | .b | 16.2 | 13.4 | 11.5 | 17.5 | 21.1 | 9.9 | 12.8 | 14.8 | 13.4 | 23.6 | 53.2 | 17.2 | 12.2 | 21.4 | 15.3 | 12.6 |
| Ghana | 2014 | 18.7 | 23.7 | 20.8 | 17.6 | 18.9 | 14.5 | 21.0 | 19.6 | 17.9 | 23.6 | 16.0 | 19.0 | 21.7 | 22.0 | 17.5 | 24.0 | 19.9 | 17.7 |
| Guatemala | 2014 | 50.7 | 43.8 | 50.2 | 50.3 | 46.2 | 51.1 | 52.3 | 42.9 | 51.9 | 55.0 | 56.2 | 50.9 | 41.2 | 54.5 | 47.7 | 44.2 | 50.0 | 54.0 |
| Guinea | 2012 | 14.9 | .b | 17.6 | 14.0 | 13.7 | 20.8 | 17.6 | 16.7 | 12.3 | 14.4 | 14.5 | 17.6 | .b | 17.4 | 13.9 | 20.2 | 14.2 | 15.1 |
| Haiti | 2016 | 11.9 | 26.3 | 15.9 | 13.9 | 6.2 | 12.8 | 19.4 | 11.3 | 13.1 | 15.9 | 14.5 | 19.0 | 26.3 | 17.9 | 6.6 | 26.9 | 16.2 | 8.8 |
| Honduras | 2011 | 50.8 | 52.9 | 56.1 | 46.8 | 39.3 | 47.7 | 58.7 | 31.9 | 49.2 | 56.9 | 59.3 | 58.9 | 43.4 | 58.6 | 44.5 | 48.0 | 52.2 | 50.4 |
| India | 2015 | 31.0 | 36.6 | 30.9 | 31.1 | 30.6 | 32.5 | 30.9 | 28.8 | 30.6 | 31.0 | 32.0 | 33.3 | 33.4 | 30.8 | 30.9 | 32.2 | 28.7 | 38.1 |
| Indonesia | 2012 | 22.4 | .b | 19.1 | 25.9 | 18.1 | 26.4 | 19.4 | 21.3 | 27.8 | 23.3 | 17.9 | 22.2 | 8.7 | 24.9 | 19.6 | 17.6 | 23.0 | 22.1 |
| Kenya | 2014 | 20.7 | 45.9 | 27.0 | 21.2 | 17.8 | 22.5 | 26.6 | 19.2 | 18.7 | 23.6 | 27.7 | 29.4 | 22.8 | 23.6 | 22.9 | 22.4 | 20.9 | 26.1 |
| Kyrgyzstan | 2012 | 17.3 | .b | 16.2 | 17.9 | Na | Na | 17.3 | 16.8 | 14.8 | 16.7 | 21.1 | 14.9 | .b | 19.9 | 15.4 | .b | 18.0 | 16.9 |
| Lesotho | 2014 | 39.6 | 38.8 | 43.8 | 37.9 | 27.5 | 32.2 | 49.0 | 34.2 | 35.4 | 40.2 | 45.0 | 43.0 | 49.6 | 43.0 | 33.5 | 49.8 | 42.3 | 32.3 |
| Liberia | 2013 | 16.5 | 11.7 | 13.8 | 17.4 | 15.3 | 15.6 | 15.3 | 18.5 | 12.5 | 16.7 | 12.3 | 17.1 | 14.8 | 14.8 | 16.0 | 19.1 | 14.5 | 15.2 |
| Malawi | 2015 | 24.1 | 33.0 | 28.8 | 24.3 | 20.6 | 24.4 | 30.1 | 25.4 | 23.8 | 23.7 | 22.5 | 29.5 | 32.4 | 32.0 | 20.4 | 37.1 | 27.0 | 18.7 |
| Mali | 2012 | 12.6 | .b | 15.3 | 11.9 | 12.0 | 13.5 | 18.1 | 9.8 | 14.1 | 10.6 | 12.4 | 15.9 | .b | 13.7 | 12.0 | 10.8 | 10.9 | 15.8 |
| Mozambique | 2015 | 20.1 | 18.6 | 19.3 | 20.1 | 18.0 | 19.1 | 25.7 | 20.3 | 18.5 | 21.2 | 17.4 | 22.5 | 14.8 | 20.3 | 20.1 | 18.4 | 18.6 | 22.9 |
| Myanmar | 2015 | 32.8 | .b | 34.5 | 32.3 | 26.8 | 32.3 | 38.4 | 25.8 | 32.7 | 30.7 | 35.9 | 43.3 | 19.2 | 37.9 | 29.4 | 20.7 | 34.5 | 32.2 |
| Namibia | 2013 | 11.2 | 10.3 | 12.6 | 9.8 | 5.0 | 10.0 | 12.7 | 11.2 | 8.3 | 13.7 | 6.2 | 17.0 | 17.5 | 7.7 | 11.9 | 15.2 | 9.1 | 12.1 |
| Nepal | 2016 | 18.2 | Na | 21.0 | 14.3 | 19.7 | 17.0 | 17.8 | 14.5 | 19.3 | 12.9 | 16.6 | 27.7 | 18.8 | 17.9 | 18.8 | 21.2 | 14.5 | 26.3 |
| Niger | 2012 | 19.1 | Na | 17.0 | 19.5 | 16.1 | 32.5 | 39.1 | 20.1 | 18.5 | 15.3 | 20.6 | 21.0 | 55.3 | 26.2 | 15.0 | 38.0 | 20.8 | 9.8 |
| Nigeria | 2013 | 17.5 | 31.4 | 25.1 | 14.1 | 6.9 | 25.1 | 26.2 | 4.2 | 15.0 | 19.1 | 23.0 | 29.9 | 18.6 | 17.9 | 17.8 | 14.0 | 15.8 | 21.7 |
| Philippines | 2017 | 22.4 | 20.1 | 21.4 | 23.0 | 3.4 | 15.7 | 24.3 | 13.5 | 20.2 | 19.9 | 30.9 | 27.2 | 13.9 | 23.9 | 21.8 | 11.9 | 20.7 | 24.2 |
| Rwanda | 2014 | 18.1 | 52.8 | 20.0 | 20.9 | 14.2 | 20.2 | 38.3 | 26.4 | 17.3 | 15.4 | 20.8 | 24.5 | 50.3 | 23.3 | 18.7 | .b | 20.5 | 20.1 |
| Senegal | 2017 | 18.9 | .b | 27.2 | 15.5 | 14.6 | 25.2 | 32.1 | 15.6 | 13.5 | 19.4 | 17.7 | 37.7 | 43.4 | 24.6 | 15.4 | 23.7 | 21.3 | 15.0 |
| Sierra Leone | 2013 | 24.3 | 22.2 | 23.0 | 24.5 | 23.0 | 25.4 | 26.8 | 22.6 | 24.1 | 25.3 | 22.3 | 26.1 | 22.9 | 27.7 | 22.2 | 27.9 | 26.4 | 19.7 |
| Tajikistan | 2012 | 27.8 | .b | 27.6 | 27.9 | 10.1 | 35.4 | 27.9 | 22.4 | 25.3 | 31.0 | 29.8 | 32.5 | .b | 32.6 | 24.7 | 11.3 | 28.7 | 27.3 |
| Tanzania | 2015 | 14.8 | 37.5 | 23.2 | 14.5 | 10.4 | 17.2 | 26.4 | 10.3 | 16.2 | 15.7 | 19.1 | 26.1 | 40.8 | 21.2 | 13.8 | 22.8 | 14.8 | 18.7 |
| Timor-Leste | 2016 | 7.0 | .b | 8.5 | 6.3 | 7.8 | 5.8 | 7.1 | 5.9 | 6.2 | 6.9 | 9.1 | 6.9 | .b | 5.6 | 8.0 | .b | 7.5 | 6.6 |
| Togo | 2013 | 18.6 | 32.2 | 20.9 | 18.8 | 16.9 | 19.9 | 23.5 | 17.1 | 21.5 | 19.7 | 18.0 | 22.1 | 29.9 | 21.7 | 17.2 | 32.4 | 18.5 | 18.7 |
| Uganda | 2016 | 17.3 | 47.1 | 28.1 | 17.9 | 15.5 | 16.9 | 31.0 | 12.8 | 17.1 | 16.9 | 25.5 | 31.6 | 34.2 | 21.9 | 18.1 | 22.2 | 17.8 | 22.7 |
| Zambia | 2013 | 16.8 | 39.1 | 20.5 | 18.7 | 15.1 | 18.4 | 22.6 | 18.0 | 16.8 | 18.8 | 25.0 | 18.9 | 40.3 | 20.0 | 17.1 | 30.8 | 19.4 | 15.6 |
| Zimbabwe | 2015 | 42.1 | 36.9 | 36.4 | 44.0 | 40.2 | 41.1 | 41.9 | 37.5 | 49.0 | 49.8 | 34.7 | 39.8 | .b | 40.4 | 42.6 | 57.5 | 39.3 | 40.9 |
| *Na (Not available) = missing values | | | |  |  |  |  |  |  |  |  |  |  |  |  |  |  |  |  |
| #.b = n <25 |  |  |  |  |  |  |  |  |  |  |  |  |  |  |  |  |  |  |  |
