# Supplementary material for: Reasons for nonuse of contraceptive methods by women with demand for contraception not satisfied: an assessment of low and middle-income countries using demographic and health surveys
Source: Reprod Health. 2019 Oct 11;16:148. doi: 10.1186/s12978-019-0805-7 (PMC6788119; doi:10.1186/s12978-019-0805-7)
Supplement: Supplementary file 9 — Additional file 9. Slope Index of Inequality (SII) calculated employing wealth index. [file 12978_2019_805_MOESM9_ESM.docx]

**Additional file 9 –** Slope Index of Inequality (SII) calculated employing wealth index.

| **Country** | **Health concerns (SII)** | **Infrequent sex (SII)** | **Other opposed (SII)** | **Respondent opposed (SII)** | **Fatalistic (SII)** | **Lack of access (SII)** | **Lack of knowledge (SII)** | **Method-related (SII)** |
| --- | --- | --- | --- | --- | --- | --- | --- | --- |
| **Demand for contraception not satisfied <30.0%** |  |  |  |  |  |  |  |  |
| Colombia | -6.9 | 18.4 | -7.0 | -7.5 | Na | -12.5 | -2.6 | Na |
| Honduras | -3.0 | 31.7 | -22.4 | -11.6 | 0.1 | -8.3 | -1.0 | -0.9 |
| Zimbabwe | 20.7 | -2.7 | -3.9 | 1.7 | -0.5 | -10.0 | 0.0 | -0.9 |
| Dominican Republic | 3.7 | 8.1 | -1.9 | -0.7 | -10.9 | 8.2 | -1.5 | -0.9 |
| Indonesia | 11.0 | -4.1 | -0.8 | -0.9 | -3.6 | -11.7 | -5.0 | 7.9 |
| Armenia | -0.8 | -24.9 | -1.7 | 9.5 | -1.9 | 0.0 | 0.3 | 1.2 |
| Cambodia | -19.0 | 25.3 | -1.6 | -1.5 | -4.8 | 0.3 | 0.1 | -0.4 |
| Guatemala | 5.7 | 14.3 | -16.3 | -0.7 | -5.2 | -7.7 | -8.5 | 1.1 |
| India | 5.4 | 5.3 | -1.4 | 3.1 | -6.7 | -2.9 | -1.5 | 0.7 |
| Namibia | 11.0 | 2.6 | -3.3 | -7.1 | -0.5 | -21.9 | -3.6 | 0.0 |
| Lesotho | -1.9 | 13.5 | -3.0 | -4.1 | 0.5 | -5.6 | 1.0 | -1.0 |
| Kenya | 14.3 | 13.6 | -8.5 | -4.8 | -1.2 | -1.7 | -6.5 | -0.6 |
| Myanmar | -10.6 | 17.9 | -2.3 | 1.4 | -3.0 | -9.0 | -1.3 | -0.9 |
| Philippines | 0.8 | 18.6 | -3.8 | 1.4 | -1.8 | -8.3 | -0.4 | -1.0 |
| Malawi | 15.8 | 4.0 | -1.1 | 0.0 | -1.3 | -1.0 | -1.5 | 0.1 |
| Congo Brazzaville | 9.6 | -2.2 | 1.4 | 2.2 | -1.2 | 2.5 | -3.7 | 0.4 |
| Rwanda | 9.1 | -2.0 | 2.4 | -1.5 | 1.9 | -2.2 | -1.0 | 1.1 |
| **Demand for contraception not satisfied 30.0-50.0%** |  |  |  |  |  |  |  |  |
| Nepal | 7.8 | 11.5 | 0.9 | -0.7 | -0.7 | -0.6 | -0.8 | 4.6 |
| Zambia | 26.9 | 5.8 | -10.2 | 0.2 | -0.5 | -8.7 | -2.3 | 0.3 |
| Kyrgyzstan | 14.8 | 1.8 | -11.3 | -7.9 | -8.5 | 1.9 | 5.4 | 1.9 |
| Tanzania | 19.1 | 16.4 | -20.2 | -7.1 | -6.1 | -6.2 | -2.7 | 0.4 |
| Ethiopia | 9.0 | 6.8 | -3.9 | -3.2 | -5.7 | -4.4 | -0.9 | 1.0 |
| Gabon | 7.5 | 10.9 | -8.1 | 8.0 | -3.5 | -1.2 | -13.0 | 4.1 |
| Uganda | 5.3 | 21.4 | -14.3 | -4.3 | -5.1 | -2.0 | 0.0 | 1.0 |
| Senegal | 4.6 | 18.1 | -5.1 | 8.5 | -11.9 | -5.6 | -2.3 | -0.2 |
| Mozambique | 4.4 | 1.4 | -11.6 | -2.3 | -5.1 | -7.8 | -12.2 | 2.5 |
| Tajikistan | 2.5 | 12.2 | 0.3 | 3.9 | -3.5 | -0.2 | -0.2 | -0.6 |
| Cameroon | 20.1 | 17.3 | -9.5 | -1.9 | -0.9 | -12.9 | -27.9 | 1.1 |
| Nigeria | 23.7 | 28.3 | -3.9 | 3.4 | -1.8 | -5.9 | -20.6 | 2.5 |
| Timor-Leste | -3.2 | 2.2 | -0.6 | 0.0 | 7.1 | -5.2 | 0.3 | -2.2 |
| **Demand for contraception not satisfied >50.0%** |  |  |  |  |  |  |  |  |
| Burundi | 1.8 | -3.0 | 9.7 | -3.5 | 1.9 | -0.6 | 0.1 | 0.5 |
| Sierra Leone | 8.3 | 2.5 | 5.4 | -2.7 | -5.7 | -6.6 | -4.1 | 1.4 |
| Ghana | 24.7 | -1.6 | -12.6 | -9.8 | -1.6 | -9.1 | -3.3 | 3.0 |
| Niger | 9.6 | 1.8 | 1.6 | 8.0 | -6.2 | -6.5 | -9.9 | -1.0 |
| Haiti | 9.9 | 7.8 | -0.6 | -2.2 | -2.0 | -3.3 | 0.3 | -0.3 |
| Congo Democratic Republic | 11.2 | 2.6 | 4.4 | -5.7 | 0.5 | -1.3 | -22.7 | 1.5 |
| Côte d’Ivoire | 16.7 | 6.5 | -0.8 | -7.1 | -2.1 | -9.3 | -22.0 | -0.2 |
| Burkina Faso | 13.6 | 10.2 | -7.0 | -2.4 | -7.4 | -13.9 | -6.1 | 0.4 |
| Liberia | 20.9 | -2.3 | 6.6 | 8.5 | -7.8 | -13.3 | -6.2 | -0.6 |
| Togo | 11.1 | 3.2 | -6.1 | 8.6 | -3.3 | -8.0 | -0.2 | -3.3 |
| Comoros | 13.6 | 11.6 | -9.5 | -3.9 | 0.1 | -0.2 | -0.2 | -2.2 |
| Benin | 21.8 | -8.6 | 5.2 | 0.3 | 1.4 | -1.6 | -9.8 | 2.8 |
| Angola | 20.7 | 1.9 | -0.2 | 9.3 | -9.4 | -3.1 | -22.4 | -0.5 |
| Mali | 9.2 | 4.3 | 1.6 | -1.0 | -2.3 | -5.9 | -3.7 | 0.1 |
| Gambia | 6.7 | 12.7 | 2.7 | -6.3 | -2.9 | -0.5 | -3.8 | 0.1 |
| Guinea | 16.5 | 1.8 | 0.3 | 8.5 | -3.6 | -9.4 | -11.3 | 0.8 |
| Chad | 9.7 | -0.1 | 2.3 | 4.7 | -1.8 | 0.8 | -7.5 | 1.2 |

*Variations in the tone of the gold color to express positive (reason most prevalent in the richest), null (absence of inequalities) and negative values (reason most prevalent in the poorest).
